# A relationship between the incremental values of area under the ROC curve and of area under the precision-recall curve

**DOI:** 10.1186/s41512-021-00102-w

**Published:** 2021-07-14

**Authors:** Qian M. Zhou, Lu Zhe, Russell J. Brooke, Melissa M. Hudson, Yan Yuan

**Affiliations:** 1 Department of Mathematics and Statistics, Mississippi State University, Mississippi State, MS USA; 2 School of Public Health, University of Alberta, Edmonton, AB Canada; 3 St. Jude Children’s Research Hospital, Memphis, TN USA

**Keywords:** Prediction performance, AUC, Area under precision-recall curve, Brier score, Proper scoring rules, Rare outcome

## Abstract

**Background:**

Incremental value (IncV) evaluates the performance change between an existing risk model and a new model. Different IncV metrics do not always agree with each other. For example, compared with a prescribed-dose model, an ovarian-dose model for predicting acute ovarian failure has a slightly lower area under the receiver operating characteristic curve (AUC) but increases the area under the precision-recall curve (AP) by 48%. This phenomenon of disagreement is not uncommon, and can create confusion when assessing whether the added information improves the model prediction accuracy.

**Methods:**

In this article, we examine the analytical connections and differences between the AUC IncV (*Δ*AUC) and AP IncV (*Δ*AP). We also compare the true values of these two IncV metrics in a numerical study. Additionally, as both are semi-proper scoring rules, we compare them with a strictly proper scoring rule: the IncV of the scaled Brier score (*Δ*sBrS) in the numerical study.

**Results:**

We demonstrate that *Δ*AUC and *Δ*AP are both weighted averages of the changes (from the existing model to the new one) in separating the risk score distributions between events and non-events. However, *Δ*AP assigns heavier weights to the changes in higher-risk regions, whereas *Δ*AUC weights the changes equally. Due to this difference, the two IncV metrics can disagree, and the numerical study shows that their disagreement becomes more pronounced as the event rate decreases. In the numerical study, we also find that *Δ*AP has a wide range, from negative to positive, but the range of *Δ*AUC is much smaller. In addition, *Δ*AP and *Δ*sBrS are highly consistent, but *Δ*AUC is negatively correlated with *Δ*sBrS and *Δ*AP when the event rate is low.

**Conclusions:**

*Δ*AUC treats the wins and losses of a new risk model equally across different risk regions. When neither the existing or new model is the true model, this equality could attenuate a superior performance of the new model for a sub-region. In contrast, *Δ*AP accentuates the change in the prediction accuracy for higher-risk regions.

**Supplementary Information:**

The online version contains supplementary material available at (10.1186/s41512-021-00102-w).

## Introduction

Risk prediction is crucial in many medical decision-making settings, such as managing disease prognosis. Numerous research has been dedicated to continually updating risk models for better prediction accuracy. For example, several papers have investigated the improvement in predicting the risk of cardiovascular disease by adding new biomarkers to the existing Framingham risk model, such as the C-reactive protein [1, 2], and more recently, a polygenic risk score [3, 4].

In some applications, an existing marker is replaced with a new marker that provides more precise information. For example, cancer treatment such as radiation can have significant long-term health consequences for cancer survivors. Prescribed radiation doses to body regions, such as the abdomen and chest, are routinely available in medical charts. But to predict the risk of an organ-specific outcome, e.g., secondary lung cancer or ovarian failure, a more precise measurement of the radiation exposure to specific organs provides better information. Radiation oncologists developed and applied algorithms to estimate these organ-specific exposures [5].

The measurement of a new marker or the more precise measurement of a known risk factor is often costly and time-consuming. Thus, it is important to verify that the new model indeed has a measurable and better prediction performance than the existing one, and thus, worth the extra resources. A number of metrics have been proposed to evaluate the incremental value (IncV) of the risk model that incorporates the new information. The IncV has primarily been discussed in settings where new markers are added to the existing risk profile [6, 7]. In this paper, the term IncV refers to the change of the prediction performance whenever an existing risk model is compared with a new one.

In medical research, the receiver operating characteristic (ROC) curve has been and remains the most popular tool for evaluating the prediction accuracy of a risk model, dating back to the 1960s when it was applied in diagnostic radiology and imaging systems [8, 9]. The area under the ROC curve (AUC) captures the discriminatory ability of a model, i.e., how well a model separates events (subjects who experience the event of interest) from non-events (subjects who are event-free). Recently, the precision-recall (PR) curve is gaining popularity [10–13]. Originated from the information retrieval community in the 1980s [14, 15], it is a relatively new tool in medical research. The area under the PR curve is called the average positive predictive value or the average precision (AP) [16–18]. Several papers suggest that the PR curve and AP are more informative than the ROC curve and AUC for evaluating the risk model’s prediction performance for an unbalanced outcome, i.e., when the event rate is low ([16, 19, 20]).

Davis and Goadrich established a one-to-one correspondence between an ROC curve and a PR curve [21]. When comparing the prediction performance of two risk models, e.g., new versus existing, the ROC curve of the new model dominates the ROC curve of the existing model if and only if the PR curve of the new model dominates the PR curve of the existing model. However, when the ROC and PR curves of the two models cross, it is not uncommon that the IncVs of AUC and AP contradict each other. Clark et al. [22] investigated two models for predicting acute ovarian failure among female childhood cancer survivors. The ovarian-dose model has a slightly lower AUC but an increased AP by about 48%, compared to the prescribed-dose model. The disagreement creates confusion in determining whether the updated risk score improves the prediction accuracy.

In this article, we investigate the analytical connection and difference between the IncVs of AUC and of AP with respect to their true values derived from the underlying data generating mechanism. Unlike previous works investigating the inconsistency between the AUC and AP mainly via simulation studies, our numerical study focuses on the true values, not estimates, of these two IncV metrics. In addition, we examine the effect of the event rate on their (dis)agreement, both analytically and numerically.

## Method

### Notation and definitions

First, we lay out the notations and define concepts that are used throughout this article. Let *D*= 0 or 1 denote a binary outcome. For studies with an event time *T*, define *D*=*I*(*T*≤*τ*) for a given prediction time period *τ*, which indicates that the outcome is time-dependent. In this article, we refer to subjects with *D*=1 as the *events* and those with *D*=0 as the *non-events*. Let *π*=*Pr*(*D*=1) denote the event rate.

#### Risk model and risk score

A risk model is a function of a set of predictors **X**=(*X*_1_,⋯,*X*_*k*−1_), which might include interaction terms and polynomial terms, to obtain the probability of *D*=1. Usually, we write this model as a regression model:

1 $$ p(\mathbf{X}) = g\left(\beta_{0}+\beta_{1}X_{1}+\cdots+\beta_{k-1}X_{k-1}\right),  $$

where *g*(·) is a smooth and monotonic *link* function, such as a logit link. For the censored event time outcomes, a risk model could be Cox’s proportional hazards model [23] or the time-specific generalized linear model [24]; both models can be expressed in the general form of Eq. (1) with modifications.

In practice, the underlying data generating mechanism is often complicated, and our working risk model in Eq. (1) is usually misspecified. Let *π*(**X**)=*Pr*(*D*=1∣**X**) denote the *true* probability of *D*=1, which is determined by the underlying distribution of *D* given **X**. Here, we refer to *π*(**X**) as the *true* risk and *p*(**X**) as the *working* risk from a working risk model. When the working risk model in Eq. (1) is misspecified, *p*(**X**)≠*π*(**X**).

The working risk *p*(**X**) can be regarded as a risk score and used to classify subjects into different risk categories. For example, given a cut-off value *c*, subjects with *p*(**X**)≤*c* are classified into the low-risk group, whereas the high-risk group consists of subjects with *p*(**X**)>*c*. In general, a risk score, denoted as *r*(**X**), can be any function of **X** that reflects how likely a subject is an event. Thus, *r*(**X**) can be a non-decreasing transformation of *p*(**X**), e.g., *r*(**X**)=*g*^−1^(*p*(**X**))=*β*_0_+*β*_1_*X*_1_+⋯+*β*_*k*−1_*X*_*k*−1_.

##### **Remark 1**

In practice, the working risk *p*(**X**) is estimated from a data sample. The estimated regression coefficients $\widehat \beta _{j},j=0,1,\cdots,k-1$, are the solution to an estimating equation: $\boldsymbol {\Psi }\left (\beta _{0},\cdots,\beta _{k-1}\right) = \sum _{i=1}^{n} \Psi \left (\beta _{0},\cdots,\beta _{k-1};D_{i},\mathbf {X}_{i}\right)$. The estimated risk given **X** is $\widehat {p}(\mathbf {X})=g(\widehat {\beta }_{0}+\widehat {\beta }_{1}X_{1} + \cdots + \widehat {\beta }_{k-1}X_{k-1})$, which is not of interest here. In this article, we investigate the predictive performance of the **population** working risk $p(\mathbf {X})=g\left (\beta _{0}^{\ast }+\beta _{1}^{\ast }X_{1} + \cdots + \beta _{k-1}^{\ast }X_{k-1}\right)$ where $\beta _{j}^{\ast }$’s are the solution of *E*_(D,**X**)_[***Ψ***(*β*_0_,⋯,*β*_*k*−1_)]=0 with the expectation taken under the true joint distribution of (*D*,**X**), and $\beta _{j}^{\ast }={\lim }_{n\rightarrow \infty }\widehat {\beta }_{j}$.

#### Accuracy measures and IncV metrics

The AUC and AP can be defined on any risk score *r*(**X**) since they are rank-based. The ROC curve is a curve of the true positive rate (TPR) versus the false positive rate (FPR). Given a cut-off value *c*, the TPR and FPR are the proportions of higher-risk score *r*(**X**)>*c* among the events and non-events respectively, i.e., TPR(*c*)=*Pr*[*r*(**X**)>*c*∣*D*=1] and FPR(*c*)=*Pr*[*r*(**X**)>*c*∣*D*=0]. The AUC can be interpreted as the conditional probability that given a pair of an event and a non-event, the event is assigned with a higher-risk score, i.e., AUC=*Pr*[*r*(**X**_*i*_)>*r*(**X**_*j*_)∣*D*_*i*_=1,*D*_*j*_=0].

The PR curve is a curve of the positive predictive value (PPV) versus the TPR. The PPV is defined as PPV(*c*)=*Pr*[*D*=1∣*r*(**X**)>*c*], the proportion of subjects with higher-risk scores that are events. The AP can be expressed as AP=*E*[PPV(*r*_1_(**X**))] [18], where *r*_1_(**X**) denotes the risk score of an event, and the expectation is taken under the distribution of *r*_1_(**X**). The AP is event-rate dependent [18]; in contrast, the AUC does not depend on *π* since it is conditional on the event status.

Let *Ψ*_*old*_ and *Ψ*_*new*_ denote an accuracy measure *Ψ* (e.g., AUC or AP) of the existing and new risk models, respectively. The IncV is defined as *ΔΨ*=*Ψ*_*new*_−*Ψ*_*old*_, which quantifies the change in *Ψ* when comparing the new model with the existing one.

### Data example

Accurate ovarian failure (AOF) is a treatment associated complication caused by ovarian exposure to radiation and chemotherapy. It is defined as permanent loss of ovarian function within 5 years of a cancer diagnosis or no menarche after cancer treatment by age 18. About 6% of female childhood cancer survivors have AOF. We evaluate and compare two recently published risk models [22] that predict AOF on an external validation dataset, the St. Jude Lifetime Cohort [25], which consists of 875 survivors with 50 AOF events.

Both models include the following risk factors: age at cancer diagnosis, cumulative dose of alkylating drugs measured using the cyclophosphamide-equivalent dose, hematopoietic stem-cell transplant, and radiation exposure. The difference between the two models is in the measurement of radiation exposure. The *prescribed-dose* model uses the prescribed radiation doses to the abdominal and pelvic regions, which are routinely available in medical charts. The *ovarian-dose* model uses the minimum of the organ-specific radiation exposure for both ovaries estimated by radiation oncologists. The equation for calculating the AOF risk using each model is developed using the Childhood Cancer Survivors Study and given in the supplementary material of Clark et al. (2020) [22].

**Fig. 1:**
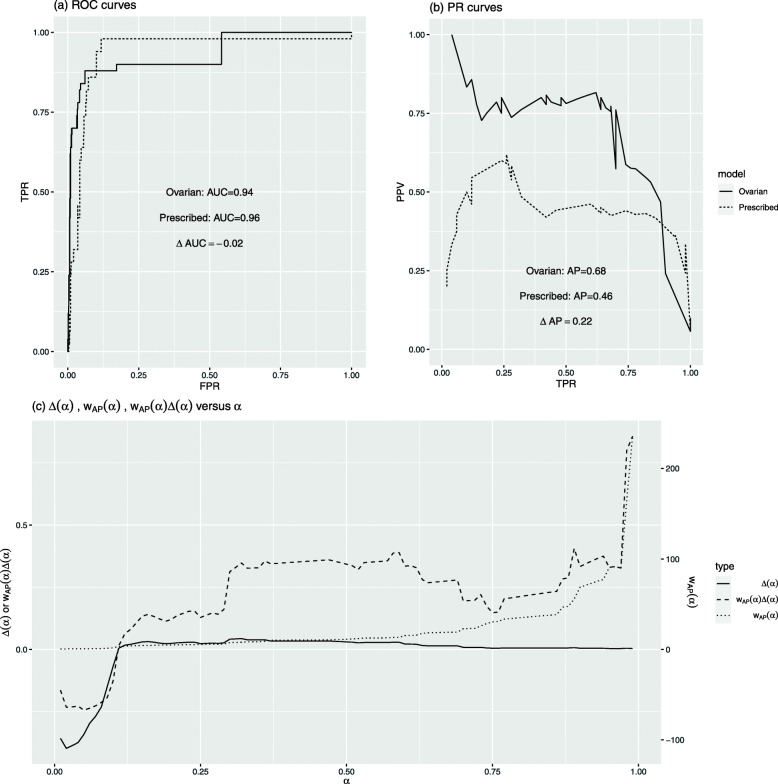
Data example: ovarian-dose vs prescribed-dose

Figure 1a and b show the ROC curves and PR curves of these two models. The estimated AUC is 0.96 for the prescribed-dose model and 0.94 for the ovarian-dose model; *Δ*AUC is estimated to be − 0.02. The estimated AP is 0.46 for the prescribed-dose model and 0.68 for the ovarian-dose model. The estimated *Δ*AP is 0.22. The estimation procedure is explained in Appendix.

Based on the *Δ*AUC, we conclude that the more expensive ovary dosimetry does not improve the prediction accuracy at all. However, based on the *Δ*AP, the ovarian-dose model clearly outperforms the prescribed-dose model. Why do these two metrics give conflicting conclusions?

### Analytical comparisons between *Δ*AUC and *Δ*AP

To answer this question, we first investigate the connections and differences between the AUC and AP using the following three hypothetical risk scores: *r*_1_,*r*_2_, and *r*_3_. We assume that all the risk scores among non-events follow a standard normal distribution, i.e., *r*_*j*_∣*D*=0∼*N*(0,1), for *j*=1,2,3. However, their distributions among events are different: (i) *r*_1_∣*D*=1∼*N*(1.8,2), (ii) *r*_2_∣*D*=1∼*N*(1.5,1.5), and (iii) *r*_3_∣*D*=1∼*N*(3,1.5).

**Fig. 2:**
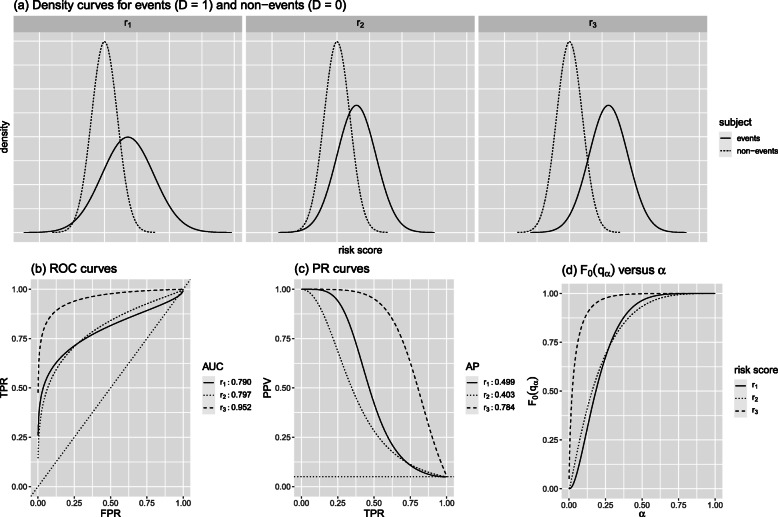
Comparison of three hypothetical risk scores *r*_1_,*r*_2_, and *r*_3_ at event rate *π*=0.05

**Fig. 3:**
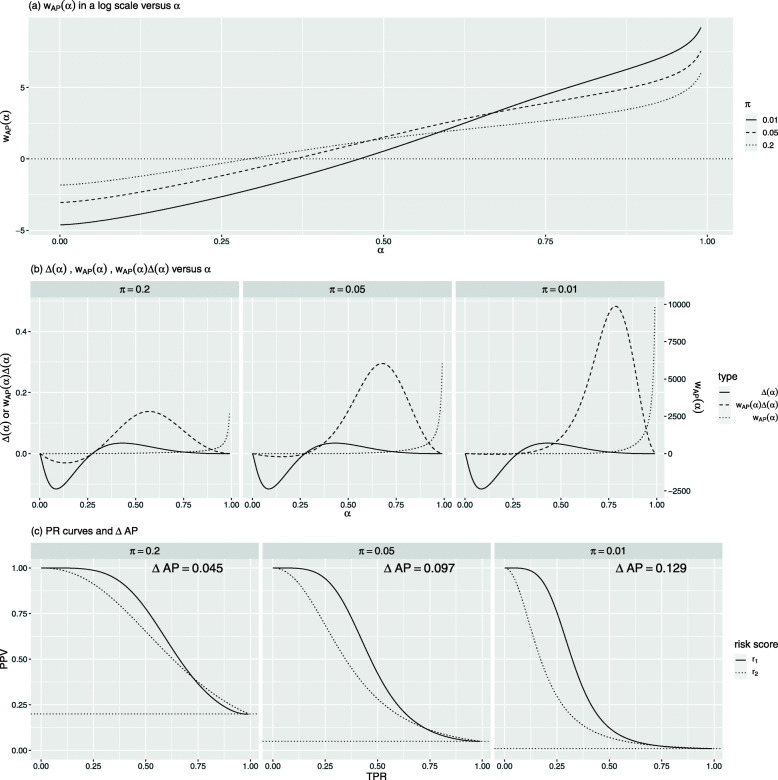
Comparison of hypothetical risk scores *r*_1_ and *r*_2_ under event rates *π*=0.2,0.05,0.01

Figure 2 presents the comparisons of these three risk scores under an event rate *π*=0.05. Figure 2a shows their density curves stratified by events and non-events. Among them, the two density curves of *r*_3_ are the most separated. Thus, the ROC and PR curves of *r*_3_ dominate those of *r*_1_ and *r*_2_ (Fig. 2b and c), and consequently, *r*_3_ has the largest AUC and AP. In contrast, the ROC and PR curves of *r*_1_ and *r*_2_ cross: *r*_2_ has a slightly larger AUC with $\text {AUC}_{r_{2}} - \text {AUC}_{r_{1}} = 0.007$, but *r*_1_ has a considerably larger AP with $\text {AP}_{r_{1}} - \text {AP}_{r_{2}} = 0.096$. Figure 3 exhibits the comparisons between *r*_1_ and *r*_2_ for three different event rates *π*=0.2, 0.05, and 0.01.

Analytically, both the AUC and AP measure the separation of the risk score distributions between events and non-events. Let *F*_1_(·) and *F*_0_(·) denote the cumulative distribution functions (CDFs) of a risk score *r*(**X**) conditional on *D*=1 (events) and *D*=0 (non-events), respectively. Let $q_{\alpha }=F_{1}^{-1}(\alpha)$ denote the *α*th quantile for the distribution *F*_1_,0≤*α*≤1. As shown in Eqs. (7) and (8) of Appendix, the AUC and AP can be expressed as functions of *F*_0_(*q*_*α*_), the proportion of non-events whose risk scores are below the *α*th quantile of the risk scores among events. The *F*_0_(*q*_*α*_) measures the separation of the two distributions *F*_1_ and *F*_0_: the larger the *F*_0_(*q*_*α*_) is at a given *α*, the more non-events having lower-risk scores, indicating a further separation between these two distributions. For example, the *F*_0_(*q*_*α*_) curve of *r*_3_ dominates those of *r*_1_ and *r*_2_ (Fig. 2d), which is consistent with the fact that *r*_3_ has the best separation between events and non-events (Fig. 2a).

Furthermore, we can express both *Δ*AUC and *Δ*AP as

2 $$ \Delta{\Psi} = \int_{0}^{1} w_{\Psi}(\alpha)\Delta(\alpha) d\alpha,\, \Psi=\text{AUC}\, \text{or}\, \text{AP},  $$

where *w*_*Ψ*_(*α*) is a weight function, and *Δ*(*α*)=*F*_*new*,0_(*q*_*new*,*α*_)−*F*_*old*,0_(*q*_*old*,*α*_), capturing how much the new working risk model changes the separation of these two distributions at a given *α*. Note that *Δ*(*α*) is independent of *π* because it is conditional on the event outcome. Thus, *Δ*AUC and *Δ*AP are weighted averages of *Δ*(*α*), but their weights are different. For *Δ*AUC,*w*_AUC_(*α*)≡1 for 0≤*α*≤1, i.e., *Δ*(*α*) is *equally* weighted. For *Δ*AP,*w*_AP_(*α*) is a function of *α* and *π* (Eq. (9) of Appendix).

To visualize how *w*_AP_(*α*) changes with *α* and *π*, we plot the *w*_AP_(*α*) in a log scale against *α* for different *π* in Fig. 3a, in the context of comparing the hypothetical risk scores *r*_1_ and *r*_2_. For any *π*,*w*_AP_(*α*) increases with *α*. This tells us that *Δ*AP assigns heavier weights to the upper-tail quantiles of the risk score, representing higher-risk regions, and lighter weights to lower-tail quantiles, representing lower-risk regions, i.e., *Δ*AP emphasizes the change of the separation in higher-risk regions. However, the change is equally weighted in *Δ*AUC since *w*_AUC_(*α*)≡1.

Additionally, *w*_AP_ is affected by *π*. When *π* is smaller, *w*_AP_(*α*) is larger for *α* values close to 1 but smaller for *α* values close to 0. This indicates that, at a lower event rate, if a risk model can better separate the two risk score distributions at the upper quantiles, it will be rewarded more; if it has a worse separation at lower quantiles, it will be penalized less.

#### Hypothetic risk scores *r*_1_ and *r*_2_ revisited

Assuming that *r*_2_ is from an existing risk model and *r*_1_ is from a new one, $\Delta (\alpha)=F_{r_{1},0}\left (q_{r_{1},\alpha }\right) - F_{r_{2},0}\left (q_{r_{2},\alpha }\right),\Delta \text {AUC} = \text {AUC}_{r_{1}} - \text {AUC}_{r_{2}}$, and $\Delta \text {AP} = \text {AP}_{r_{1}}-\text {AP}_{r_{2}}$. As shown in Fig. 3b, *Δ*(*α*)>0 for large *α*, and *Δ*(*α*)<0 for small *α*. It indicates that compared to *r*_2_,*r*_1_ has a better separation for the upper quantiles of the risk score but worse for lower quantiles. With equal weighting, *Δ*AUC is equivalent to the area under *Δ*(*α*) curve over its entire range. Since the area above 0 is approximately the same as the area below 0, *Δ*AUC≈0. As mentioned earlier, *Δ*AUC is invariant for different *π*. Thus, *Δ*AUC=−0.007 (Fig. 2b) for all three *π* values.

For *Δ*AP, the *r*_1_’s upper-tail better performance is weighted more than its lower-tail worse performance, which explains *Δ*AP is all positive for the three *π* values (Fig. 3c). Additionally, when *π* gets smaller, the better separation of *r*_1_ at the upper quantiles is rewarded more, and meanwhile, its worse separation at lower quantiles is penalized less. Thus, even though *Δ*(*α*) stays the same across different *π*,*Δ*AP increases as *π* decreases (Fig. 3c).

#### Data example revisited

Let *Δ*(*α*)=*F*_ovarian,0_(*q*_*ovarian*,*α*_)−*F*_prescribed,0_(*q*_*prescribed*,*α*_). Figure 1c plots the estimated *Δ*(*α*),*w*_AP_(*α*), and *w*_AP_(*α*)*Δ*(*α*). It shows that the estimated *Δ*(*α*)>0 for *α*>10%, whereas the prescribed-dose model performs better with the estimated *Δ*(*α*)<0 when *α*<10%. It suggests that compared to the prescribed-dose model, the ovarian-dose model separates the events and non-events better among individuals predicted to be at a higher risk. Overall, under the estimated *Δ*(*α*) curve, the area below zero is slightly larger than the area above zero. Thus, the estimated *Δ*AUC is negative but close to zero. This indicates that these two models have comparable performance in terms of *Δ*AUC.

However, the estimated *Δ*AP rewards the superior performance of the ovarian-dose model at the upper quantiles with large weights, and thus, it is positive and sizable. Clark et al. [22] created four risk groups: low (< 5%), medium-low (5% to < 20%), medium (20% to < 50%), and high risk (≥ 50%). The ovarian-dose model classifies 37 individuals (out of 875) as high risk, among which 30 (81%) experienced AOF, while the prescribed-dose model predicted 13 individuals at high risk, with 6 (46%) AOF events. This again confirms that the ovarian-dose model is better at identifying the AOF events.

##### Comparison with Brier score.

Since both the AUC and AP are rank-based, they are semi-proper scoring rules: the true model has the maximum AUC and AP among all the models, but a misspecified risk model and the true model can have the same AUC and AP when they rank the subjects’ risks in the same order. We decide to compare these two metrics with the Brier score (BrS), the only strictly proper scoring rule.

The BrS is the expected squared difference between the binary outcome *D* and the working risk *p*(**X**), i.e., BrS=*E*_(D,**X**)_{[*D*−*p*(**X**)]^2^}. The BrS is minimized at the true model, i.e., *p*(**X**)=*π*(**X**). A non-informative model, assigning the event rate to every subject, i.e., *p*(**X**)≡*π*, leads to the maximum BrS value *π*(1−*π*). A *scaled Brier score* (sBrS) is defined as sBrS=1−BrS/[*π*(1−*π*)], ranging from 0 and 1, with larger values indicating better performance [26].

###### **Remark 2**

Although the BrS cannot be directly expressed as a function of *F*_0_(*q*_*α*_), it is closely related to the two distributions *F*_1_ and *F*_0_. Specifically, it can be written as

$$\begin{array}{*{20}l} \text{BrS} & = E\left\{\left[1-p(\mathbf{X})\right]^{2}\mid D=1\right\}\pi \\ & + E\left\{\left[p(\mathbf{X})\right]^{2}\mid D=0\right\}(1-\pi). \end{array} $$

The first expectation is the mean squared prediction error (MSPE) of the working risk *p*(**X**) for events, determined by the distribution *F*_1_, whereas the second expectation is the MSPE for non-events, determined by the distribution *F*_0_. Both MSPEs can be expressed as the sum of the variance of *p*(**X**) and its squared bias from 1 for events and from 0 for non-events. A smaller BrS can result from one, or a combination, of the following: (i) the mean of *p*(**X**)for events closer to 1, (ii) the mean of *p*(**X**)for non-events closer to 0, (iii) less variation in *p*(**X**) for events or non-events or both. All of these lead to a further separation of the two distributions: *F*_1_ and *F*_0_.

Let *Δ*sBrS denoted the IncV of sBrS. The sBrS is estimated to be 0.23 for the prescribed-dose model and 0.50 for the ovarian-dose model, and *Δ*sBrS is estimated to be 0.27. Thus, similar to *Δ*AP,*Δ*sBrS favors the ovarian-dose model.

Why are *Δ*sBrS and *Δ*AP consistent in this example? Figure S1 of the supplementary material shows the histogram of the predicted risk $\widehat p_{i}$ from each model among the AOF and non-AOF individuals. For the non-AOF individuals, the risk score distributions of these two models are similar. Consequently, the mean and variance of $\widehat p_{i}$ for both models are also similar: the mean is 0.033 for the ovarian-dose model and 0.042 for the prescribed-dose model; their variances are both about 0.0053. The MSPE for the ovarian-dose model is 0.0064, slightly lower than 0.0071 for the prescribed-dose model.

For the AOF events, the risk score distribution of the ovarian model has a heavier right tail. This indicates that the ovarian-dose model pushes more AOF events to the high-risk group. As a result, the mean of $\widehat p_{i}$ for the ovarian-dose model is 0.48, much closer to 1 than 0.23 for the prescribed-dose model. The variance is 0.10 for the ovarian-dose model and 0.023 for the prescribed-dose model. The MSPE of the ovarian-dose model is 0.367, much smaller than 0.613 of the prescribed-dose model. Combining the MSPEs for events and non-events weighted by their respective proportions, the estimated BrS for the ovarian-dose model is 0.027, which is smaller than 0.042, the estimated BrS for the prescribed-dose model.

This data example illustrates a comparison of the three IncV metrics: *Δ*AUC,*Δ*AP, and *Δ*sBrS. Next, we expand the comparison via a numerical study.

### Numerical study

As we are interested in the *true values* of the IncV for the population working risk (described in Remark 1), not in the IncV estimates from a sample, we do not use simulation studies; there are no data or samples involved. The numerical study in this section evaluates the IncV of adding a marker, denoted by *Y*, to a model with an existing marker, denoted by *X*. The true value of each IncV metric is directly derived from the distributional assumptions described below.

Let the markers *X* and *Y* be independent standard normal random variables. Given the values of these two markers, a binary outcome *D* follows a Bernoulli distribution with the probability of *D*=1 via the following model:

3 $$ \begin{aligned} \pi(X,Y) & = Pr(D=1\mid X,Y) \\ & = \Phi(\beta_{0}+\beta_{1}X+\beta_{2}Y+\beta_{3}XY), \end{aligned}  $$

where *Φ*(·) is the CDF of a standard normal distribution. Given *X* and *Y*, *π*(*X*,*Y*) is the *true* risk. The true model in Eq. (3) includes an interaction between *X* and *Y*, indicating the effect of *X* on the risk changes with the value of *Y*, and vice versa.

Typically, in practice, none of the working models are the true model. Having this in mind, we compare the following two misspecified working models: (i) *one-marker model*: *p*(*X*)=*Φ*(*γ*_0_+*γ*_1_*X*), and (ii) *two-marker model*: *p*(*X*,*Y*)=*Φ*(*γ*_0_+*γ*_1_*X*+*γ*_2_*Y*).

Here, we consider different values of *β*_1_,*β*_2_,*β*_3_ and *π*: *β*_1_=0.3,0.4,⋯,0.9,1,*β*_2_=0.3,0.4,⋯,0.9,1,*β*_3_=− 0.5,− 0.4,⋯,− 0.1,0.1,⋯,0.4,0.5 (excluding 0), and *π*=0.01, 0.05, 0.1, 0.2, 0.5. Each combination of (*β*_1_,*β*_2_,*β*_3_,*π*) values is referred to as a scenario. Given a scenario, the value of *β*_0_ can be derived. In the supplementary material, we explain how to obtain the value of *β*_0_ and calculate the true values of AUC, AP, and sBrS of the one-marker and two-marker models as well as the true values of the IncV metrics.

## Results

We compare the three IncV metrics based on the following two aspects: (1) size and range, and (2) agreement. A desirable IncV metric should be sensitive to the change in the predictive performance. If a new model improves the prediction accuracy, an IncV should have a sizable positive value. It should also be able to reflect a performance *deterioration* with a sizable negative value. If an IncV is often close to 0, we might question its utility in supporting decision-making. As mentioned earlier, inconsistency among different accuracy metrics is often encountered. Thus, we are also interested in the agreement among the three IncV metrics.

### Size and range

**Fig. 4:**
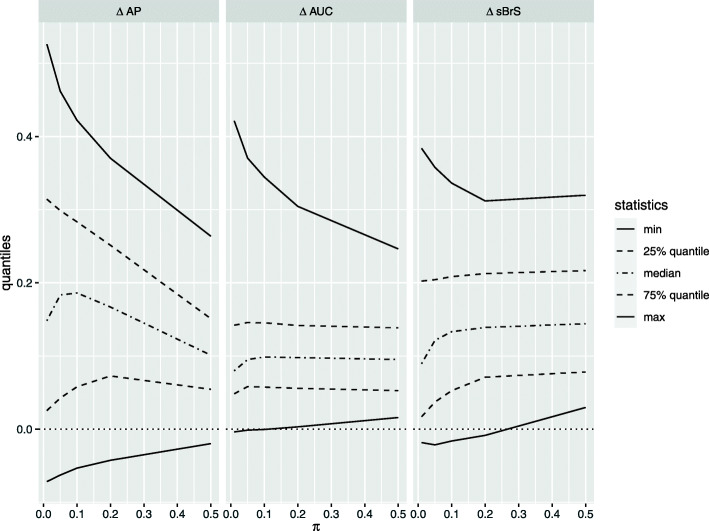
Summary statistics of *Δ*AUC,*Δ*AP, and *Δ*sBrS versus different event rates *π*

Figure 4 plots the summary statistics (minimum, 25% quantile, median, 75% quantile, and maximum) of the three IncV metrics under different event rates. *Δ*AP has the widest range, followed by *Δ*sBrS, and *Δ*AUC has the narrowest range. This difference between *Δ*AUC and *Δ*AP is more evident for a lower event rate. For example, under *π*=0.01, the inter-quartile range (IQR) and median of *Δ*AUC are both 0.07. In contrast, the IQR of *Δ*AP is much wider, with a range of about 0.41 and a median of 0.21.

In addition, *Δ*AUC is negative in less than 1% of the scenarios (29 out of 3200). Furthermore, when it is negative, the value is very close to 0, which indicates that *Δ*AUC cannot distinguish between a useless marker and a harmful marker [27]. On the other hand, *Δ*AP is negative in about 12% of the scenarios (389 out of 3200), with a much larger size.

As *π* changes, the range of *Δ*AP varies the most among the three IncV metrics, whereas the quartiles of *Δ*AUC remain almost constant. As *π* increases, the ranges of all the IncV metrics get narrower and closer to each other. When *π*=0.5, both *Δ*AUC and *Δ*AP range from 0.015 to 0.25 with a median of 0.089, and *Δ*sBrS ranges from 0.019 to 0.32 with a median of 0.12.

### Agreement

#### Correlation

We calculate the Pearson correlation between each pair of the IncV metrics under each *π* (Table 1). *Δ*AP and *Δ*sBrS are highly correlated for all values of *π*. As *π* increases, their correlation decreases from about 1 (*π*=0.01) to 0.84 (*π*=0.5), but the correlations of *Δ*AUC with the other two IncV metrics increases with *π*. When *π*=0.01,*Δ*AUC and *Δ*sBrS are negatively correlated and their correlation − 0.11 is the smallest among the three pairs; when *π*=0.5, they are the highest positively correlated. We also show the scatter plots of each pair under different *π* in Figure S7 (supplementary material).

**Table 1:** Pearson correlation and concordance measure of each pair of the IncV metrics for different event rates *π*

Comparison	*π*=0.01	*π*=0.05	*π*=0.1	*π*=0.2	*π*=0.5
Pearson correlation					
*Δ*sBrSvs *Δ*AP	0.995	0.992	0.986	0.971	0.837
*Δ*sBrS vs *Δ*AUC	− 0.111	0.262	0.479	0.718	0.932
*Δ*AUC vs *Δ*AP	− 0.086	0.296	0.505	0.708	0.888
Concordance					
*Δ*sBrS vs *Δ*AP	0.931	0.922	0.897	0.856	0.922
*Δ*sBrS vs *Δ*AUC	0.659	0.750	0.828	0.928	1.000
*Δ*AUC vs *Δ*AP	0.591	0.672	0.725	0.784	0.922

#### Concordance

The sign of an IncV metric is often used to decide whether the new model is more accurate than the existing one. Positive IncVs favor the new model, while negative or zero values favor the existing one. Here, we define a concordance measure, which quantifies the consistency of the conclusions reached by a pair of IncV metrics.

Take *Δ*AP and *Δ*sBrS as an example. Under a scenario, we call the pair concordant if both are > 0 or ≤ 0. If one is > 0 and the other is ≤ 0, the pair is discordant. The measure of concordance is defined as the proportion of scenarios where the pair is concordant minus the proportion of scenarios where it is discordant. For instance, when *π*=0.01,*Δ*AP and *Δ*sBrS are concordant in about 97% of the total 640 scenarios (i.e., all the combinations of *β*_1_,*β*_2_, and *β*_3_ values at each *π*) and discordant in about 3%. Thus, the concordance measure is 0.93 with a roundoff error.

Table 1 reports the concordance for all three pairs of the IncV metrics under each *π*. The results are similar to those above for the Pearson correlation. When *π* is small, such as 0.01, 0.05, and 0.1, *Δ*AP and *Δ*sBrS are the most concordant; when *π*=0.2 or 0.5, *Δ*AUC and *Δ*sBrS are the most concordant. *Δ*AUC and *Δ*AP are the least concordant for all values of *π*.

When *π* is close to 0.5, the three IncV metrics tend to agree. Using any of them, we would very likely reach the same conclusion about whether the new model is more accurate. However, when the event rate is low, i.e., for a rare outcome, *Δ*AUC can be inconsistent with both *Δ*sBrS and *Δ*AP.

### *Δ*AUC versus *Δ*AP in selected scenarios

**Fig. 5:**
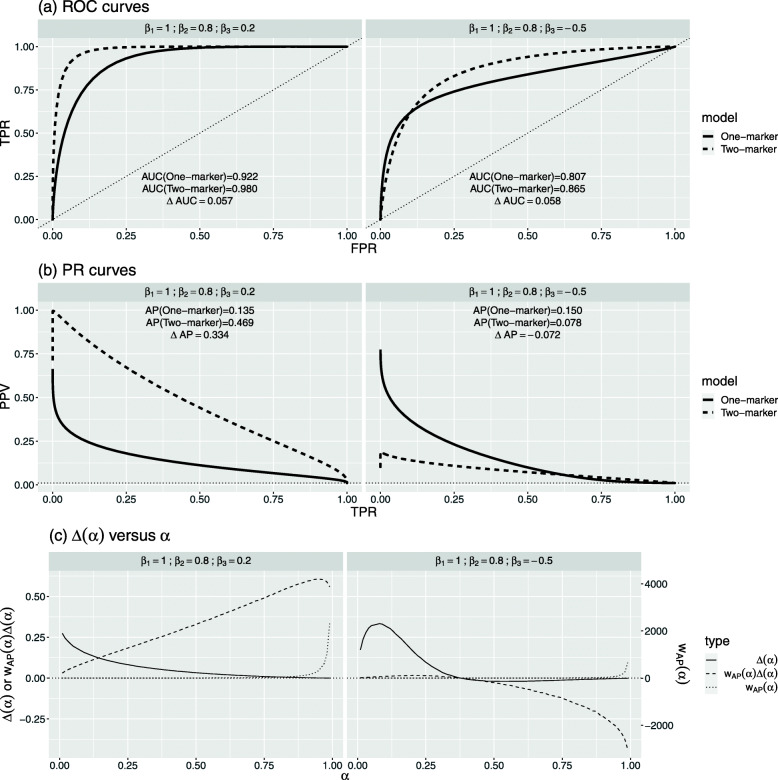
Comparison of two scenarios at event rate *π*=0.01: similar *Δ*AUC but different *Δ*AP

**Fig. 6:**
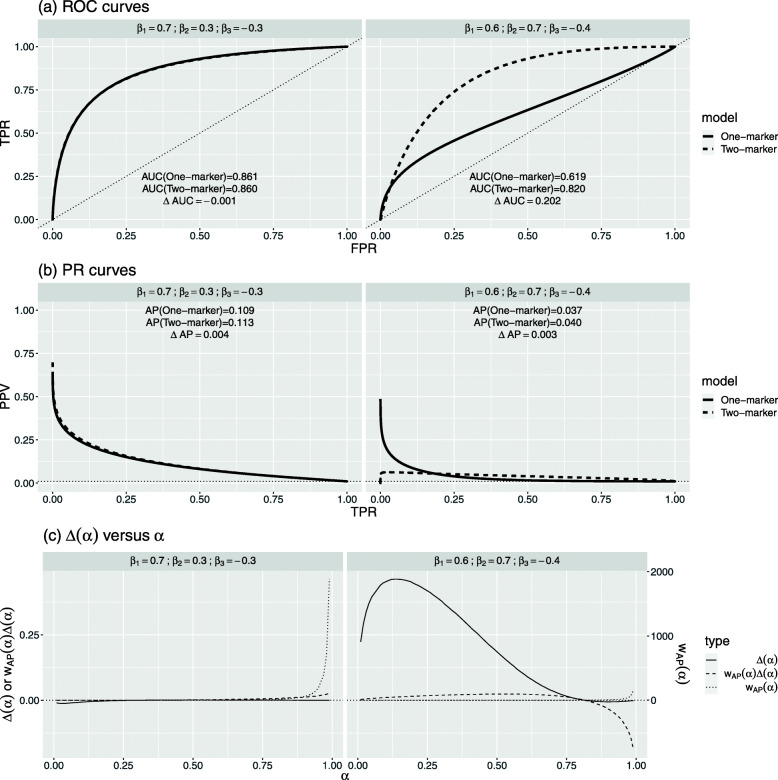
Comparison of two scenarios at event rate *π*=0.01: similar *Δ*AP but different *Δ*AUC

Next, we single out four scenarios for an in-depth comparison between *Δ*AUC and *Δ*AP at *π*=0.01. The first two scenarios have similar *Δ*AUC but different *Δ*AP (Fig. 5), whereas the next two have similar *Δ*AP but different *Δ*AUC (Fig. 6).

#### Similar *Δ*AUC but different *Δ*AP

The two scenarios are (i) *β*_1_=1,*β*_2_=0.8, and *β*_3_=0.2, and (ii) *β*_1_=1,*β*_2_=0.8, and *β*_3_=−0.5. In both cases, *Δ*AUC is around 0.06, but *Δ*AP is 0.33 for scenario (i) and − 0.072 for scenario (ii).

In scenario (i), both the ROC and PR curves of the two-marker model dominate those of the one-marker model, respectively. This indicates that the two-marker model is better at each point, and consequently, *Δ*(*α*) is positive throughout (Fig. 5c). In this case, both *Δ*AUC and *Δ*AP are positive. However, the size of *Δ*AP 0.33 is much larger than *Δ*AUC 0.06, due to the large weight *w*_AP_(*α*) at the upper quantiles (Fig. 5c).

In scenario (ii), both the two ROC curves and PR curves cross, and *Δ*(*α*) is below zero for upper quantiles and above zero for lower quantiles (Fig. 5c). This implies that the two-marker model can better separate between events and non-events for lower-risk regions, but not for higher-risk regions. As a result, *Δ*AUC and *Δ*AP are conflicting. *Δ*AUC is positive because the area under *Δ*(*α*) curve above zero is larger than that below zero. However, *Δ*AP is negative, as it weights the below-zero *Δ*(*α*) heavily.

#### Similar *Δ*AP but different *Δ*AUC

The next two scenarios are (iii) *β*_1_=0.7,*β*_2_=0.3, and *β*_3_=− 0.3, and (iv) *β*_1_=0.6,*β*_2_=0.7, and *β*_3_=−0.4. In both cases, *Δ*AP values are almost 0, but *Δ*AUC is approximately 0 for scenario (iii) and 0.202 for scenario (iv).

In scenario (iii), the two ROC curves and the two PR curves are almost identical. This indicates that adding the new marker does not change the separation of the distributions of the risk score between events and non-events. It is also reflected in Fig. 6c where the entire *Δ*(*α*) curve almost overlaps with the zero line. Thus, both *Δ*AUC and *Δ*AP are close to zero. This is an example of both metrics agreeing that the new marker is “useless.”

In scenario (iv), although the two-marker model makes poorer predictions for higher-risk regions, its prediction is significantly better for the rest. Thus, *Δ*AUC is positive and sizable. However, since *Δ*AP weights heavily on higher-risk regions, the improvement on the majority is offset by the worse performance at the upper quantiles, which leads to a close-to-zero *Δ*AP.

### What if the two-marker model is the true model, i.e., *β*_3_ = 0?

Figures S8 and S9 in the supplementary material examine this question and show the scatter plots and plots of the summary statistics of *Δ*AUC,*Δ*AP, and *Δ*sBrS for different *π*. As expected, all the IncVs are positive. For a smaller *π*,*Δ*AP ranges wider than *Δ*AUC does. As *π* increases, these two metrics get closer to each other. When *π*=0.5,*Δ*sBrS has the widest range.

Since all the IncVs are positive, their concordance is all 1. Table S1 (supplementary material) lists the Pearson correlation between each pair of the IncV metrics, which are all positive. When *π* is small, *Δ*sBrS is more strongly correlated with *Δ*AP than with *Δ*AUC. As *π* increases, all three IncV metrics are strongly correlated with each other.

## Discussion

Pepe et al. (2013) proved that, when one of the two working models is the true model, the hypothesis *H*_0_:*p*(*X*,*Y*)=*p*(*X*) is equivalent to the hypotheses of no improvement in the accuracy measures such as the AUC, net reclassification index (NRI), or integrated discrimination improvement (IDI) [6]. In their setting, the ROC and PR curves never cross. However, our paper focuses on situations where neither working model is the true model, and the two curves might cross. When they cross each other, the above equivalence among the hypotheses does not hold, and it implies that one of the two models outperforms the other at non-overlapping risk regions. This could lead to the disagreement between *Δ*AUC and *Δ*AP.

*Δ*AUC has been criticized for being insensitive to the contribution from an added marker [28]. According to our analysis, the insensitivity is likely a result of its equal treatment across different risk regions, and thus, it often fails to reflect the “local” improvement or deterioration of the new risk model. In the AOF example, the ovarian-dose model demonstrates its superiority in higher-risk regions. However, this advantage disappears in *Δ*AUC, which takes a simple average over the ovarian-dose model’s wins in higher-risk regions and its losses in lower-risk regions.

Similarly, if we consider a curve of negative predictive values (NPV, the proportion of non-events among subjects having a lower-risk score than a cut-off value) versus specificity (1− FPR), following our derivation of AP, the area under this curve can be expressed as *E*[*NPV*(*r*_0_(**X**))] where *r*_0_(**X**) denotes the risk score of a non-event subject. We can regard this quantity to be the average NPV. Similar to *Δ*AP, the IncV of the average NPV, *Δ*aNPV, can be expressed as a weighted average of the change in the separation of the risk score distributions between events and non-events. However, its weight is larger for lower-tail quantiles of the risk score, indicating the average NPV emphasizes on the accuracy of lower-risk regions.

Assessing the change in prediction accuracy is important in investigating the potential of a new marker (or a new measurement for an existing marker) [29]. However, neither *Δ*AUC or *Δ*AP considers the cost and benefit associated with the clinical utility of risk prediction [29, 30]. Going back to the AOF example, should more expensive ovary dosimetry be used for predicting AOF because it identifies more AOF cases? Unfortunately, both *Δ*AP and *Δ*AUC are insufficient to answer this question. Vickers and Elkin [29] proposed a net benefit and decision curve analysis for evaluating the clinical value of a risk model. The net benefit is defined as $NB(p_{t}) = \pi _{1} \text {TPR}(p_{t})- (1-\pi _{1})\text {FPR}(p_{t})\frac {p_{t}}{1-p_{t}}$, quantifying the net benefit for subjects who are treated based on the rule that the risk probability is above the threshold value *p*_*t*_.

We can express the above net benefit as a function of PPV: $NB(p_{t})=Pr\left [p(\mathbf {X})>p_{t}\right ] \frac {PPV(p_{t})-p_{t}}{1-p_{t}}$. Here, *Pr*[*p*(**X**)>*p*_*t*_] is the proportion of subjects who receive the treatment among the population, and $\frac {PPV(p_{t})-p_{t}}{1-p_{t}}$ quantifies the expected net benefit given that a subject is treated. The net benefit is regarded as the scaled “average benefit per prediction” [31, 32], and thus, $\frac {PPV(p_{t})-p_{t}}{1-p_{t}}$ is the average benefit per *treated subject*. Thus, *ΔNB*(*p*_*t*_) is determined by the change of the proportion of treated subjects between the two models and *ΔPPV*(*p*_*t*_). The analytical relationship between *ΔNB* and other IncV metrics such as *ΔPPV*(*p*_*t*_) and *Δ*AP is worth further investigation.

Because the ranges of AUC, AP, and sBrS are different, the domains of their IncV metrics are also different: *Δ*AUC∈[− 0.5,0.5],*Δ*AP∈[*π*−1,1−*π*], and *Δ*sBrS∈[− 1,1]. It may be worthwhile to consider rescaling these IncV metrics to range from − 1 and 1. Alternatively, an IncV metric can be defined as a ratio such as *Ψ*_*new*_/*Ψ*_*old*_.

The AUC is conditional on the binary outcome, and consequently, only depends on the respective risk score distributions among the events and non-events. Thus, it can be estimated from either a prospective cohort study or a case-control study. In contrast, the AP is conditional on the risk score obtained at baseline. Besides the risk score distributions, the AP also depends on the event rate, and thus, it has previously only been possible to estimate from cohort studies, but not from case-control studies. However, if one can acquire the information on the event rate from a previous cohort study or from surveillance data, the AP can be estimated via combining an estimated or assumed event rate with the risk score distributions of events and non-events estimated from the case-control study using the derived expression of the AP (see Eq. (8) in Appendix).

## Conclusion

In this article, we investigated the disagreement between two IncV metrics *Δ*AUC and *Δ*AP when neither the existing nor the new risk model is the true model. We showed that they are intrinsically connected; both can be expressed as an average of *Δ*(*α*), a quantity characterizing the change in the separation of the risk score distributions between events and non-events when comparing an existing risk model to a new one. However, *Δ*AP is a weighted average, with weights monotonically increasing as the risk score increases, whereas *Δ*AUC is a simple average of the change. Due to this difference, they do not always agree with each other; the lower the event rate is, the more these two metrics disagree. In addition, compared to *Δ*AUC,*Δ*AP has a wider range and is subsequently more sensitive to the contribution from new information added to the existing risk model. Via the numerical study, we also show that *Δ*AP and *Δ*sBrS are highly consistent, but the correlation of *Δ*AUC and *Δ*sBrS transitions from a positive correlation to a negative one as the event rate decreases.

## Appendix

### Estimation of AUC, AP, and sBrS for binary outcomes

Suppose that the data $\mathfrak {D}=\{(D_{i},\mathbf {X}_{i}),i=1,\cdots,n\}$ is collected from *n* subjects. Let $\widehat {p}_{i}$ denoted the estimated risk, described in Remark 1. Let $\widehat {r}_{i}$ be a risk score, which is a non-decreasing transformation of $\widehat {p}_{i}$. The AUC and AP are estimated using $\widehat {r}_{i}$ by the following nonparametric estimators

$$\widehat{\text{AUC}} = \frac{\sum_{i=1}^{n} \sum_{j=1}^{n} I(D_{i}=1)I(D_{j}=0)I(\widehat{r}_{i}>\widehat{r}_{j})}{\sum_{i=1}^{n} \sum_{j=1}^{n} I(D_{i}=1)I(D_{j}=0)}, $$

and

$$\begin{aligned} {}\widehat{\text{AP}} = \!\frac{\sum_{i=1}^{n} \left[I\left(D_{i}=1\right)\sum_{j=1}^{n} I\left(D_{j}=1\right) I\left(\widehat{r}_{j}>\widehat{r}_{i}\right)/ \sum_{j=1}^{n} I\left(\widehat{r}_{j} > \widehat{r}_{i}\right)\right]}{\sum_{i=1}^{n} I\left(D_{i}=1\right)}. \end{aligned} $$

The BrS can be estimated using $\widehat {p}_{i}$ by $\widehat {\text {BrS}} = n^{-1}\sum _{i=1}^{n} \left (D_{i}-\widehat {p}_{i}\right)^{2}$. The event rate is estimated as $\widehat {\pi } = n^{-1}\sum _{i=1}^{n} D_{i}$. Then the sBrS is estimated as $\widehat {\text {sBrS}} = 1 - \widehat {\text {BrS}}/\left [\widehat {\pi }\left (1-\widehat {\pi }\right)\right ]$.

### Derivation of AUC and AP

Let *π*=*Pr*(*D*=1) be the event rate, and *r*(**X**)=*r*_**X**_ be a risk score. Let *F*(*c*)=*Pr*(*r*_**X**_≤*c*) denote its cumulative distribution function (CDF) for the entire population, and *F*_1_(*c*)=*Pr*(*r*_**X**_≤*c*∣*D*=1) and *F*_0_(*c*)=*Pr*(*r*_**X**_≤*c*∣*D*=0) denote its CDFs for events and non-events, respectively.

The TPR, FPR, and PPV are

4 $$\begin{array}{*{20}l}  \text{TPR}(c) & = Pr(r_{\text{ \textbf{X}}} > c \mid D=1) = 1 - F_{1}(r) \end{array} $$

5 $$\begin{array}{*{20}l}  \text{FPR}(c) & = Pr(r_{\text{ \textbf{X}}}>c \mid D=0) = 1-F_{0}(r) \end{array} $$

6 $$\begin{array}{*{20}l}  \text{PPV}(c) & = Pr(D=1 \mid r_{\text{ \textbf{X}}}>c) \\ &= \frac{Pr(D=1, r_{\text{ \textbf{X}}}>c)}{Pr(r_{\text{ \textbf{X}}}>c)} = \frac{\pi \left[1-F_{1}(c)\right]}{1-F(c)} \\ & = \frac{\pi \left[1-F_{1}(c)\right]}{\pi \left[1-F_{1}(c)\right] + (1-\pi)\left[1-F_{0}(c)\right]} \end{array} $$

where 1−*F*(*c*)=*π*[1−*F*_1_(*c*)]+(1−*π*)[1−*F*_0_(*c*)].

**AUC** is the area under the ROC curve, which can be expressed as

$$\begin{aligned} \text{AUC} & = \int_{\infty}^{-\infty} \text{TPR}(c) d \text{FPR}(c) \\ & = 1 - \int_{\infty}^{-\infty} \text{FPR}(c) d \text{TPR}(c) \\ & = \int_{\infty}^{-\infty} \left[1- \text{FPR}(c) \right] d \text{TPR}(c), \end{aligned} $$

because $\int _{\infty }^{-\infty } d \text {TPR}(c) = 1$. Using the expressions in Eqs. (4) and (5), we have

$$\text{AUC} = \int_{\infty}^{-\infty} F_{0}(c) d[1-F_{1}(c)] = \int_{-\infty}^{\infty} F_{0}(c)dF_{1}(c). $$

Let $q_{\alpha }=F_{1}^{-1}(\alpha)$ be the *α*th quantile of the *F*_1_ distribution, i.e., *F*_1_(*q*_*α*_)=*α*. Thus, let *c*=*q*_*α*_, and we have

7 $$ \text{AUC} = \int_{0}^{1} F_{0}(q_{\alpha}) d\alpha.  $$

**AP** is the area under the PR curve, which can be expressed as

$$\text{AP} = \int_{\infty}^{-\infty} \text{PPV}(c)d\text{TPR}(c). $$

Using the expressions in Eqs. (4) and (6), we have

$$\begin{array}{*{20}l} {}\text{AP} & = \int_{\infty}^{-\infty} \frac{\pi F_{1}(c)}{\pi F_{1}(c) + (1-\pi)F_{0}(c)} d[1-F_{1}(c)] \\ & = \int_{-\infty}^{\infty} \frac{\pi \left[1-F_{1}(c)\right]}{\pi \left[1-F_{1}(c)\right] + (1-\pi)\left[1-F_{0}(c)\right]} dF_{1}(c)\\ & = \int_{-\infty}^{\infty} \left\{\frac{\pi \left[1-F_{1}(c)\right] + (1-\pi)\left[1-F_{0}(c)\right]}{\pi \left[1-F_{1}(c)\right]}\right\}^{-1} dF_{1}(c) \\ & = \int_{-\infty}^{\infty} \left\{1 + \frac{1-\pi}{\pi}\frac{1-F_{0}(c)}{1-F_{1}(c)}\right\}^{-1} dF_{1}(c). \end{array} $$

Again, let *c*=*q*_*α*_, we have

8 $$ \text{AP} = \int_{0}^{1} \left\{1+\frac{\pi^{-1}-1}{1-\alpha}\left[1-F_{0}(q_{\alpha})\right]\right\}^{-1}d\alpha.  $$

### Weight *w*_AP_ in *Δ*AP

Let AP_*old*_ and *α*_*new*_ denote the AP of the existing and new models:

$$\begin{array}{*{20}l} \text{AP}_{old} & = \int_{0}^{1} \left\{1+\frac{\pi^{-1}-1}{1-\alpha}\left[1-F_{old,0}\left(q_{old,\alpha}\right)\right]\right\}^{-1}d\alpha,\\ \text{AP}_{new} & = \int_{0}^{1} \left\{1+\frac{\pi^{-1}-1}{1-\alpha}\left[1-F_{new,0}\left(q_{new,\alpha}\right)\right]\right\}^{-1}d\alpha. \end{array} $$

Thus, with arithmetic operations, the IncV of AP can be expressed as

$$\begin{array}{*{20}l} \Delta \text{AP} & = \text{AP}_{old} - \text{AP}_{new} \\ & = \int_{0}^{1} w_{\text{AP}}(\alpha) \left[F_{new,0}(q_{new,\alpha}) - F_{old,0}(q_{old,\alpha})\right] d\alpha, \end{array} $$

where

9 $${} w_{\text{AP}}(\alpha) \!= \!\frac{\frac{\pi^{-1}-1}{1-\alpha}}{\left[\!1\,+\,(\pi^{-1}\,-\,1)\!\frac{1-F_{new,0}(q_{new,\alpha})}{1-\alpha}\!\right]\!\left[\!1\,+\,(\pi^{-1}\,-\,1)\frac{1-F_{old,0}(q_{old,\alpha})}{1-\alpha}\!\right]}.  $$

It is a function of *α* and *π*. It also depends on *F*_*new*,0_(*q*_*new*,*α*_) and *F*_*old*,0_(*q*_*new*,*α*_). In general, *F*_0_(*q*_*α*_)≥*α* because the density curve for non-events is to the left of that for events. Thus, how the weight changes with *α* and *π* is mainly determined by the numerator (*π*^−1^−1)/(1−*α*). However, when *π* and *α* are fixed, larger values of *F*_*old*,0_(*r*_*old*,*α*_) or *F*_*new*,0_(*r*_*new*,*α*_) or both, i.e., better performance of at least one model, lead to larger weights.

## Supplementary Information

**Additional file 1** Supplementary material. The supplementary material includes (i) the histograms of the predicted AOF risk obtained from the prescribed-dose model and ovarian-dose model for individuals with and without AOF, respectively, (ii) the procedure of obtaining the true values of the IncV metrics under the distributional assumptions of the numerical study, (iii) the results of the numerical study scenarios in which neither of the working risk models is the true model, including plots of the values of each IncV metric for all the scenarios under different event rates, and the scatter plots of each pair of the IncV metrics, and (iv) the results for the scenarios where the two-marker model is the true model, including plots of the values of each IncV metric for all the scenarios under different event rates, plots of their summary statistics, and a table listing the Pearson correlation of each pair of the IncV metrics. (PDF file)

## Data Availability

The R code for the numerical study and analyzing the data example is available in https://github.com/michellezhou2009/IncVAUCAP.

## Abbreviations

AOF: Acute ovarian failure
AP: Average positive predictive value or average precision
AUC: Area under the ROC curve
BrS: Brier score
CDF: Cumulative distribution function
FPR: False positive rate
IDI: Integrated discrimination improvement
IncV: Incremental value
NB: Net benefit
NRI: Net reclassification index
PPV: Positive predictive value
PR: Precision-recall
ROC: Receiver operating characteristic
sBrS: Scaled Brier score
TPR: True positive rate

## References

[1] Cook NR, Buring JE, Ridker PM (2006). The effect of including c-reactive protein in cardiovascular risk prediction models for women. Ann Intern Med.

[2] Buckley DI, Fu R, Freeman M, Rogers K, Helfand M (2009). C-reactive protein as a risk factor for coronary heart disease: a systematic review and meta-analyses for the us preventive services task force. Ann Intern Med.

[3] Mosley JD, Gupta DK, Tan J, Yao J, Wells QS, Shaffer CM, Kundu S, Robinson-Cohen C, Psaty BM, Rich SS (2020). Predictive accuracy of a polygenic risk score compared with a clinical risk score for incident coronary heart disease. JAMA.

[4] Elliott J, Bodinier B, Bond TA, Chadeau-Hyam M, Evangelou E, Moons KG, Dehghan A, Muller DC, Elliott P, Tzoulaki I (2020). Predictive accuracy of a polygenic risk score–enhanced prediction model vs a clinical risk score for coronary artery disease. JAMA.

[5] Howell RM, Smith SA, Weathers RE, Kry SF, Stovall M (2019). Adaptations to a generalized radiation dose reconstruction methodology for use in epidemiologic studies: an update from the md anderson late effect group. Radiat Res.

[6] Pencina MJ, D’Agostino Sr RB, D’Agostino Jr RB, Vasan RS (2008). Evaluating the added predictive ability of a new marker: from area under the roc curve to reclassification and beyond. Stat Med.

[7] Pepe MS, Kerr KF, Longton G, Wang Z (2013). Testing for improvement in prediction model performance. Stat Med.

[8] Zweig MH, Campbell G (1993). Receiver-operating characteristic (roc) plots: a fundamental evaluation tool in clinical medicine. Clin Chem.

[9] Pepe MS (2003). The Statistical Evaluation of Medical Tests for Classification and Prediction.

[10] Badawi O, Liu X, Hassan E, Amelung PJ, Swami S (2018). Evaluation of icu risk models adapted for use as continuous markers of severity of illness throughout the icu stay. Crit Care Med.

[11] Chaudhury S, Brookes KJ, Patel T, Fallows A, Guetta-Baranes T, Turton JC, Guerreiro R, Bras J, Hardy J, Francis PT (2019). Alzheimer’s disease polygenic risk score as a predictor of conversion from mild-cognitive impairment. Transl Psychiatry.

[12] Tang M, Hu P, Wang C-F, Yu C-Q, Sheng J, Ma S-J (2019). Prediction model of cardiac risk for dental extraction in elderly patients with cardiovascular diseases. Gerontology.

[13] Xiao J, Ding R, Xu X, Guan H, Feng X, Sun T, Zhu S, Ye Z (2019). Comparison and development of machine learning tools in the prediction of chronic kidney disease progression. J Transl Med.

[14] Raghavan V, Bollmann P, Jung GS (1989). A critical investigation of recall and precision as measures of retrieval system performance. ACM Trans Inf Syst (TOIS).

[15] Manning CD, Schütze H (1999). Foundations of Statistical Natural Language Processing.

[16] Yuan Y, Su W, Zhu M (2015). Threshold-free measures for assessing the performance of medical screening tests. Front Public Health.

[17] Su W, Yuan Y, Zhu M (2015). A relationship between the average precision and the area under the roc curve. Proceedings of the 2015 International Conference on The Theory of Information Retrieval.

[18] Yuan Y, Zhou QM, Li B, Cai H, Chow EJ, Armstrong GT (2018). A threshold-free summary index of prediction accuracy for censored time to event data. Stat Med.

[19] Ozenne B, Subtil F, Maucort-Boulch D (2015). The precision–recall curve overcame the optimism of the receiver operating characteristic curve in rare diseases. J Clin Epidemiol.

[20] Saito T, Rehmsmeier M (2015). The precision-recall plot is more informative than the roc plot when evaluating binary classifiers on imbalanced datasets. PloS One.

[21] Davis J, Goadrich M (2006). The relationship between precision-recall and roc curves. Proceedings of the 23rd International Conference on Machine Learning. ICML ’06.

[22] Clark RA, Mostoufi-Moab S, Yasui Y, Vu NK, Sklar CA, Motan T, Brooke RJ, Gibson TM, Oeffinger KC, Howell RM, Smith SA, Lu Z, Robison LL, Chemaitilly W, Hudson MM, Armstrong GT, Nathan PC, Yuan Y (2020). Predicting acute ovarian failure in female survivors of childhood cancer: a cohort study in the childhood cancer survivor study (ccss) and the st jude lifetime cohort (sjlife). Lancet Oncol.

[23] Cox DR (1972). Regression models and life tables. JR Stat Soc B.

[24] Uno H, Cai T, Tian L, Wei L (2007). Evaluating prediction rules for t-year survivors with censored regression models. J Am Stat Assoc.

[25] Hudson MM, Ness KK, Nolan VG, Armstrong GT, Green DM, Morris EB, Spunt SL, Metzger ML, Krull KR, Klosky JL (2011). Prospective medical assessment of adults surviving childhood cancer: study design, cohort characteristics, and feasibility of the st. jude lifetime cohort study. Pediatr Blood Cancer.

[26] Steyerberg EW, Vickers AJ, Cook NR, Gerds T, Gonen M, Obuchowski N, Pencina MJ, Kattan MW (2010). Assessing the performance of prediction models: a framework for some traditional and novel measures. Epidemiol (Cambridge, Mass).

[27] Kattan MW, Gerds TA (2018). The index of prediction accuracy: an intuitive measure useful for evaluating risk prediction models. Diagn Prognostic Res.

[28] Pepe MS, Janes H, Longton G, Leisenring W, Newcomb P (2004). Limitations of the odds ratio in gauging the performance of a diagnostic, prognostic, or screening marker. Am J Epidemiol.

[29] Vickers AJ, Elkin EB (2006). Decision curve analysis: a novel method for evaluating prediction models. Med Dec Making.

[30] Shah NH, Milstein A, Bagley SC (2019). Making machine learning models clinically useful. JAMA.

[31] Baker SG, Kramer BS (2007). Peirce, youden, and receiver operating characteristic curves. Am Stat.

[32] Baker SG, Schuit E, Steyerberg EW, Pencina MJ, Vickers A, Moons KG, Mol BW, Lindeman KS (2014). How to interpret a small increase in auc with an additional risk prediction marker: decision analysis comes through. Stat Med.

