# Supplementary material for: A relationship between the incremental values of area under the ROC curve and of area under the precision-recall curve
Source: Diagn Progn Res. 2021 Jul 14;5:13. doi: 10.1186/s41512-021-00102-w (PMC8278775; doi:10.1186/s41512-021-00102-w)
Supplement: Supplementary file 1 — Additional file 1 Supplementary material. The supplementary material includes (i) the histograms of the predicted AOF risk obtained from the prescribed-dose model and ovarian-dose model for individuals with and without AOF, respectively, (ii) the procedure of obtaining the true values of the IncV metrics under the distributional assumptions of the numerical study, (iii) the results of the numerical study scenarios in which neither of the working risk models is the true model, including plots of the values of each IncV metric for all the scenarios under different event rates, and the scatter plots of each pair of the IncV metrics, and (iv) the results for the scenarios where the two-marker model is the true model, including plots of the values of each IncV metric for all the scenarios under different event rates, plots of their summary statistics, and a table listing the Pearson correlation of each pair of the IncV metrics. (PDF file) [file 41512_2021_102_MOESM1_ESM.pdf]

Supplementary Material of "A relationship between the incremental values of area under the ROC curve and of area under the precision-recall curve"

Qian M. Zhou Zhe Lu Russell J. Brooke Melissa M Hudson
Yan Yuan

The supplementary material includes

- the histograms of the predicted AOF risk for the data example,
- the procedure of obtaining the true values of the IncV metrics under the distributional assumptions of the numerical study scenarios in which neither of the working risk models is the true model, and
- the results of the numerical study scenarios in which the two-marker model is the true model.

1 Results of AOF data example

Figure S1 shows the histograms of the predicted AOF risk obtained from the ovarian-dose model and the prescribed-dose model among individuals with and without AOF, respectively.

2 Numerical study: Calculation of IncV parameters

All the calculations are implemented by the R software (R Core Team, 2020). The integrals are calculated by the R package *pracma* (Borchers, 2019). We use the R function *uniroot* of the R package *stat* for obtaining the solution to one-dimensional equations, and use the R package *nleqslv* (Hasselman, 2018) for solving multi-dimensional equations.

Distributional assumptions. Let $\pi = Pr(D = 1)$. The two markers X and Y are independent standard normal distribution. Given X and Y, the binary outcome D follows a Bernoulli distribution with the probability of $D = 1$ given as

$$Pr(D = 1 \mid X, Y) = \Phi(\beta_0 + \beta_1 X + \beta_2 Y + \beta_3 XY) \triangleq \pi(X, Y; \boldsymbol{\beta}),$$

where $\boldsymbol{\beta} = (\beta_0, \beta_1, \beta_2, \beta_3)'$, and Φ is the cumulative distribution function of a standard normal distribution. In the numerical study, we consider two misspecified working risk models: (i) *one-marker model*: $p(X) = \Phi(\gamma_0 + \gamma_1 X)$, and (ii) *two-marker model*: $p(X, Y) = \Phi(\gamma_0 + \gamma_1 X + \gamma_2 Y)$.

A combination of the $(\beta_1, \beta_2, \beta_3, \pi)$ values is referred to as a scenario. Given a scenario, we first obtain the value of β_0. Second, we obtain the regression coefficients (γ_0, γ_1) for the one-marker model and $(\gamma_0, \gamma_1, \gamma_2)$ for the two-marker model. Third, we calculate the accuracy measures of each model. The IncV parameters are calculated by $\Delta\Psi = \Psi_{M_2} - \Psi_{M_1}$, where Ψ denotes an accuracy metric, such as the AUC, AP, and sBrS, M_1 denotes the one-marker model, and M_2 denotes the two-marker model.

Calculation of β_0 value. The event rate π can be expressed as

$$\pi = Pr(D = 1) = \int_{-\infty}^{\infty} \int_{-\infty}^{\infty} \pi(x, y; \boldsymbol{\beta})\phi(x)\phi(y)dxdy, \tag{1}$$

where $\phi(\cdot)$ is the probability density function (PDF) of the standard normal distribution. Given the values of $(\beta_1, \beta_2, \beta_3, \pi)$, the right hand side of the equation (1) is a function of β_0. By solving this equation, we can obtain the value of β_0.

Calculation of the true values of the regression parameters for working risk models. We can express the working risk from each model by $\Phi(\boldsymbol{\gamma}'\mathbf{U})$. For the one-marker model, $\mathbf{U} = (1, X)'$ and $\boldsymbol{\gamma} = (\gamma_0, \gamma_1)'$; for the two-marker model, $\mathbf{U} = (1, X, Y)'$ and $\boldsymbol{\gamma} = (\gamma_0, \gamma_1, \gamma_2)'$. The *population* values (i.e., limiting values) of the regression parameters $\boldsymbol{\gamma}$ are the solution to the following estimating equation (EE)

$$\psi(\boldsymbol{\gamma}) = E_{X,Y,D}\left\{\mathbf{U}\frac{\phi(\boldsymbol{\gamma}'\mathbf{U})}{\Phi(\boldsymbol{\gamma}'\mathbf{U})\left[1 - \Phi(\boldsymbol{\gamma}'\mathbf{U})\right]}\left[D - \Phi(\boldsymbol{\gamma}'\mathbf{U})\right]\right\} = 0, \tag{2}$$

where the expectation is taken with respect to the joint distribution of (X, Y, D). This EE (2) can be re-written as

$$\begin{aligned}\psi(\boldsymbol{\gamma}) &= E_{X,Y}\left\{\mathbf{U}\frac{\phi(\boldsymbol{\gamma}'\mathbf{U})}{\Phi(\boldsymbol{\gamma}'\mathbf{U})\left[1 - \Phi(\boldsymbol{\gamma}'\mathbf{U})\right]}E\left[D - \Phi(\boldsymbol{\gamma}'\mathbf{U}) \mid X, Y\right]\right\} \\ &= \int_{-\infty}^{\infty}\int_{-\infty}^{\infty}\mathbf{u}\frac{\phi(\boldsymbol{\gamma}'\mathbf{u})}{\Phi(\boldsymbol{\gamma}'\mathbf{u})\left[1 - \Phi(\boldsymbol{\gamma}'\mathbf{u})\right]}\left[\pi(x, y; \boldsymbol{\beta}) - \Phi(\boldsymbol{\gamma}'\mathbf{u})\right]\phi(x)\phi(y)dxdy.\end{aligned}$$

Given the values of $\boldsymbol{\beta}$, the EE is a function of only $\boldsymbol{\gamma}$. Thus, the solution to this EE gives the population values of $\boldsymbol{\gamma}$.

Calculation of the true values of AUC, AP, and sBrS. Given the values of $\boldsymbol{\gamma}$, we can obtain the working risk $\Phi(\boldsymbol{\gamma}'\mathbf{U})$ for any given values of $\mathbf{U}$. The Brier score can be expressed as

$$\text{BrS} = E\left\{\pi(X, Y; \boldsymbol{\beta})\left[1 - \pi(X, Y; \boldsymbol{\beta})\right]\right\} + E\left\{\left[\pi(X, Y; \boldsymbol{\beta}) - \Phi(\boldsymbol{\gamma}'\mathbf{U})\right]^2\right\},$$

which can be calculated by

$$\text{BrS} = \int_{-\infty}^{\infty}\int_{-\infty}^{\infty}\left\{\pi(X, Y; \boldsymbol{\beta})\left[1 - \pi(X, Y; \boldsymbol{\beta})\right] + \left[\pi(X, Y; \boldsymbol{\beta}) - \Phi(\boldsymbol{\gamma}'\mathbf{U})\right]^2\right\}\phi(x)\phi(y)dxdy.$$

Thus, $\text{sBrS} = 1 - \text{BrS}/[\pi(1 - \pi)]$.

Both the AUC and AP are invariant under any risk score that is a non-decreasing transformation of the working risk. Thus, we consider the risk score $r = \boldsymbol{\gamma}'\mathbf{U}$. As shown in Appendix A.1, both the AUC and AP depends on the distribution of r for events and non-events. Let $f(c)$ denote the

(unconditional) PDF of r. Note that for the one-marker model, r follows a normal distribution with mean γ_0 and variance γ_1^2, i.e., $f(c) = \frac{1}{|\gamma_1|}\phi\left(\frac{c-\gamma_0}{\gamma_1}\right)$. For the two-marker model, the PDF of r can be obtained by $f(c) = f_{r|X}(c|x)\phi(x)$ or $f_{r|Y}(c|y)\phi(y)$, where $f_{r|X}(c|x) = \frac{1}{|\gamma_2|}\phi\left(\frac{c-\gamma_0-\gamma_1 x}{\gamma_2}\right)$ is the conditional PDF of r given $X = x$, and $f_{r|Y}(c|y) = \frac{1}{|\gamma_1|}\phi\left(\frac{c-\gamma_0-\gamma_2 y}{\gamma_1}\right)$ is the conditional PDF of R given $Y = y$.

The PDF of r given $D = 1$ is

$$
\begin{aligned}
f_1(c) &= \frac{\int_{-\infty}^{\infty} Pr(D=1 \mid r=c, X=x) f_{r|X}(c \mid x)\phi(x)dx}{Pr(D=1)} \\
&= \frac{\int_{-\infty}^{\infty} \pi\left(x, \frac{c-\gamma_0-\gamma_1 x}{\gamma_2}; \boldsymbol{\beta}\right) f_{r|X}(c \mid x)\phi(x)dx}{\pi}
\end{aligned} \tag{3}
$$

or

$$
\begin{aligned}
f_1(c) &= \frac{\int_{-\infty}^{\infty} Pr(D=1 \mid r=c, Y=y) f_{r|Y}(c \mid y)\phi(y)dy}{Pr(D=1)} \\
&= \frac{\int_{-\infty}^{\infty} \pi\left(\frac{c-\gamma_0-\gamma_2 y}{\gamma_1}, y; \boldsymbol{\beta}\right) f_{r|Y}(c \mid y)\phi(y)dy}{\pi}.
\end{aligned} \tag{4}
$$

Note that for the one-marker model, we can only use the equation (4) because r only depends on X. Also, $f_{r|Y}(c \mid y) = f(c) = \frac{1}{|\gamma_1|}\phi\left(\frac{c-\gamma_0}{\gamma_1}\right)$. Consequently, the CDF of r given $D = 1$ is $F_1(c) = \int_{-\infty}^{c} f_1(z)dz$.

Similarly, the PDF of r given $D = 0$ is

$$
f_0(c) = \frac{\int_{-\infty}^{\infty} \left[1 - \pi\left(x, \frac{c-\gamma_0-\gamma_1 x}{\gamma_2}; \boldsymbol{\beta}\right)\right] f_{r|X}(c \mid x)\phi(x)dx}{1-\pi}
$$

or

$$
f_0(c) = \frac{\int_{-\infty}^{\infty} \left[1 - \pi\left(\frac{c-\gamma_0-\gamma_2 y}{\gamma_1}, y; \boldsymbol{\beta}\right)\right] f_{r|Y}(c \mid y)\phi(y)dy}{1-\pi}.
$$

And the CDF of r given $D = 0$ is $F_0(c) = \int_{-\infty}^{c} f_0(z)dz$.

The AUC is given as

$$
\text{AUC} = \int_{-\infty}^{\infty} F_0(c) f_1(c)dc,
$$

and the AP is given as

$$
\text{AP} = \int_{-\infty}^{\infty} \left[1 + \left(\pi^{-1} - 1\right)\frac{1-F_0(c)}{1-F_1(c)}\right]^{-1} f_1(c)dc.
$$

3 Results of the numerical study

In this section,

- Figures S2 - S7 include the results for the scenarios in which neither of the working risk models is the true model. Figure S2 - S6 plot the values of the ΔAUC, ΔAP, and ΔsBrS for all the scenarios under each of the five event rates. Figure S7 includes the scatter plots of each pair of the IncV metrics by different event rates.
- Figure S8, Figure S9, and Table S1 include the results of the scenarios in which the two-marker model is the true model, i.e., $\beta_3 = 0$, for different event rates. The results include plots of the values of each IncV metric for all the scenarios and different event rates, plots of their summary statistics, and the Pearson correlation of each pair of the IncV metrics.

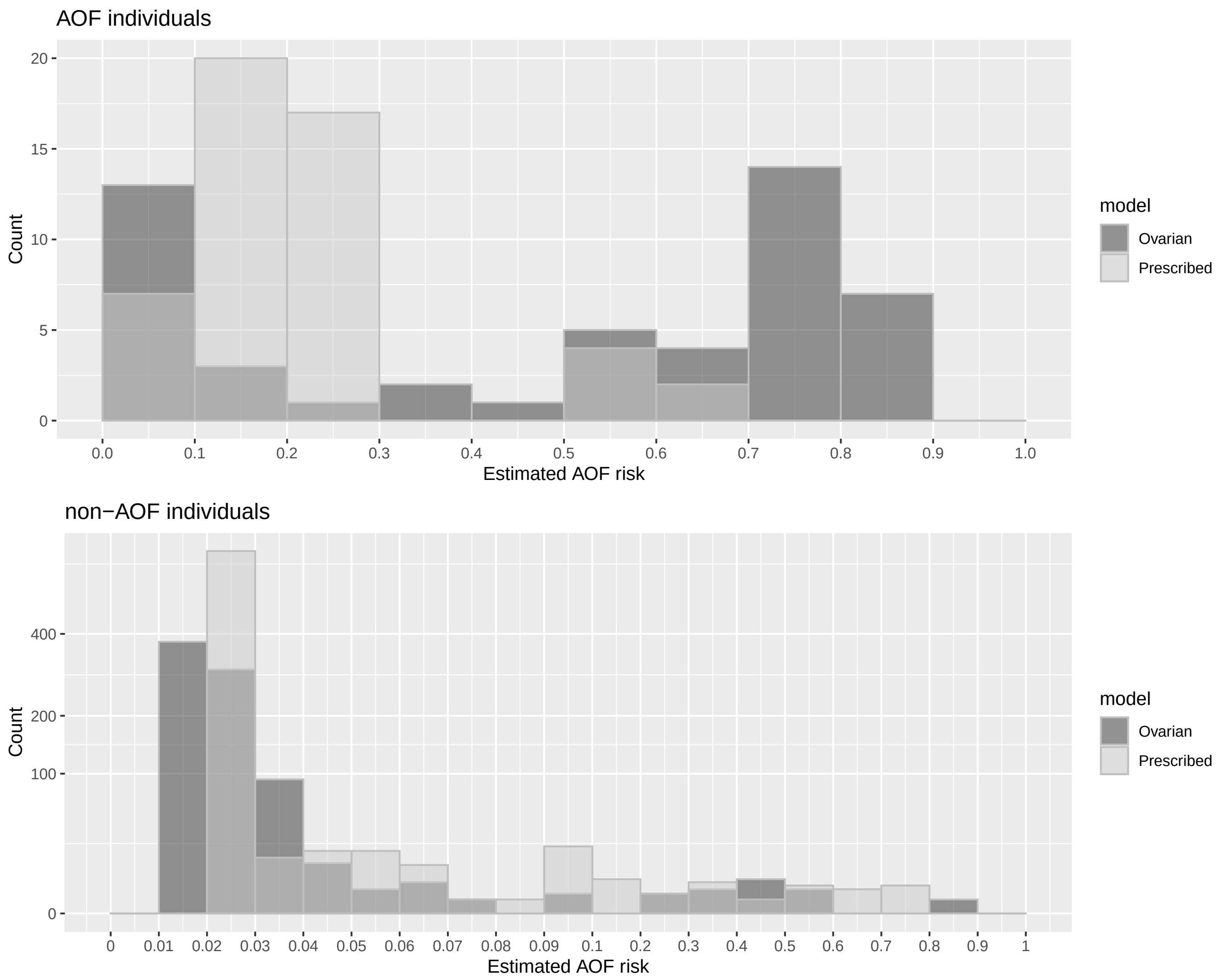

Figure S1: Results of Data Example: Histograms of the predicted AOF risk obtained from the prescribed-dose model and ovarian-dose model for individuals with and without AOF, respectively

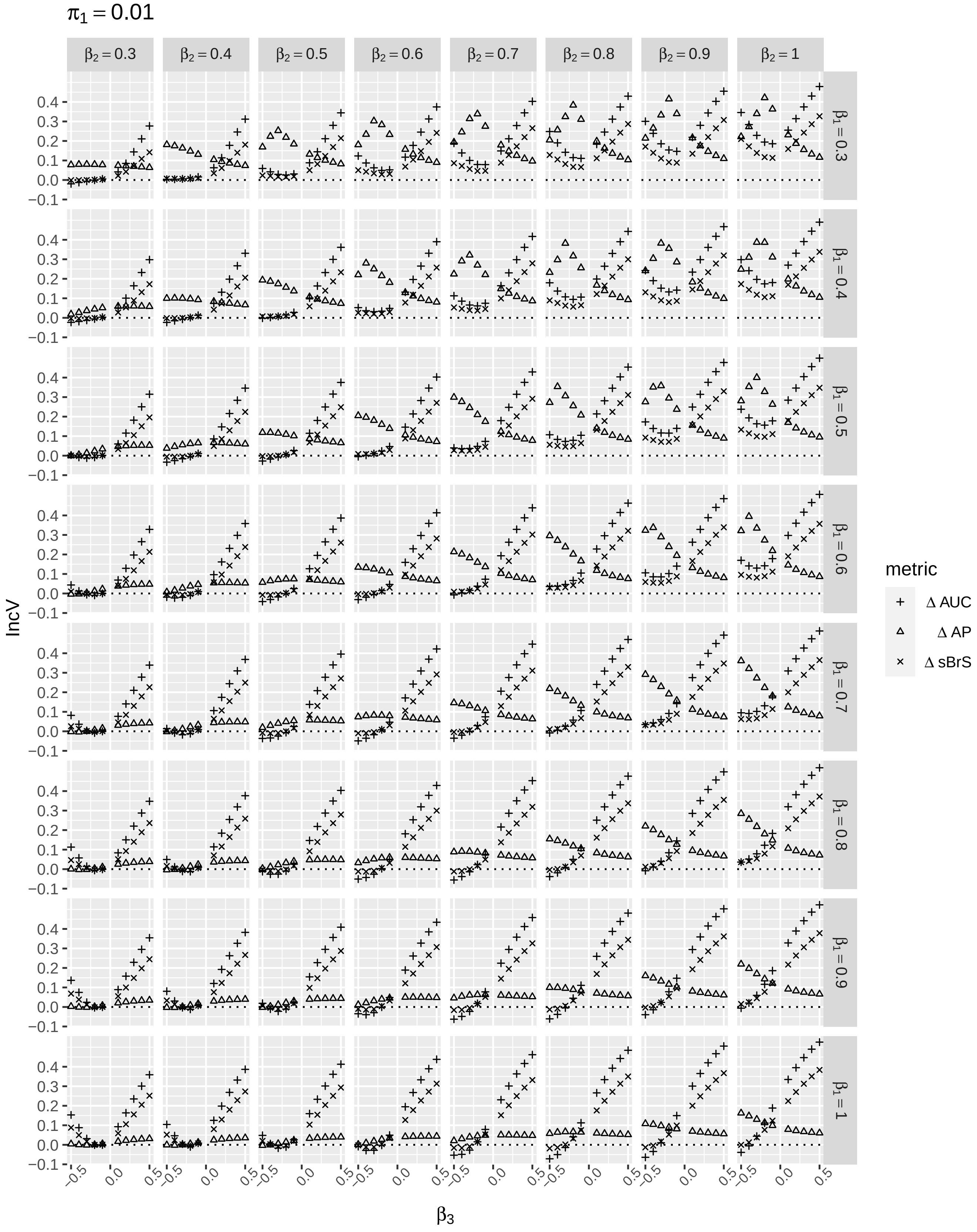

Figure S2: The ΔAUC, ΔAP, and ΔsBrS under the event rate $\pi = 0.01$.

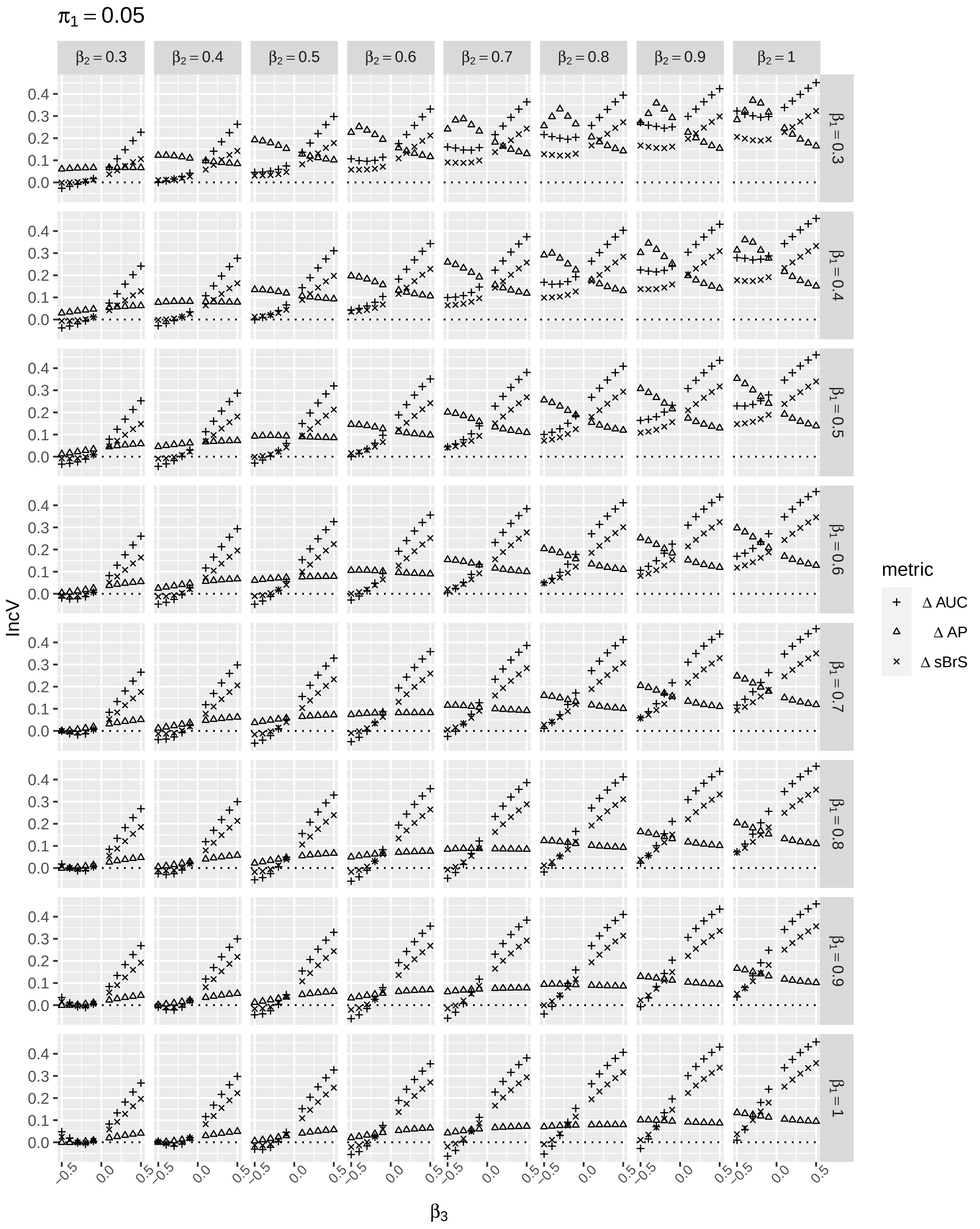

Figure S3: The ΔAUC, ΔAP, and ΔsBrS under the event rate $\pi = 0.05$.

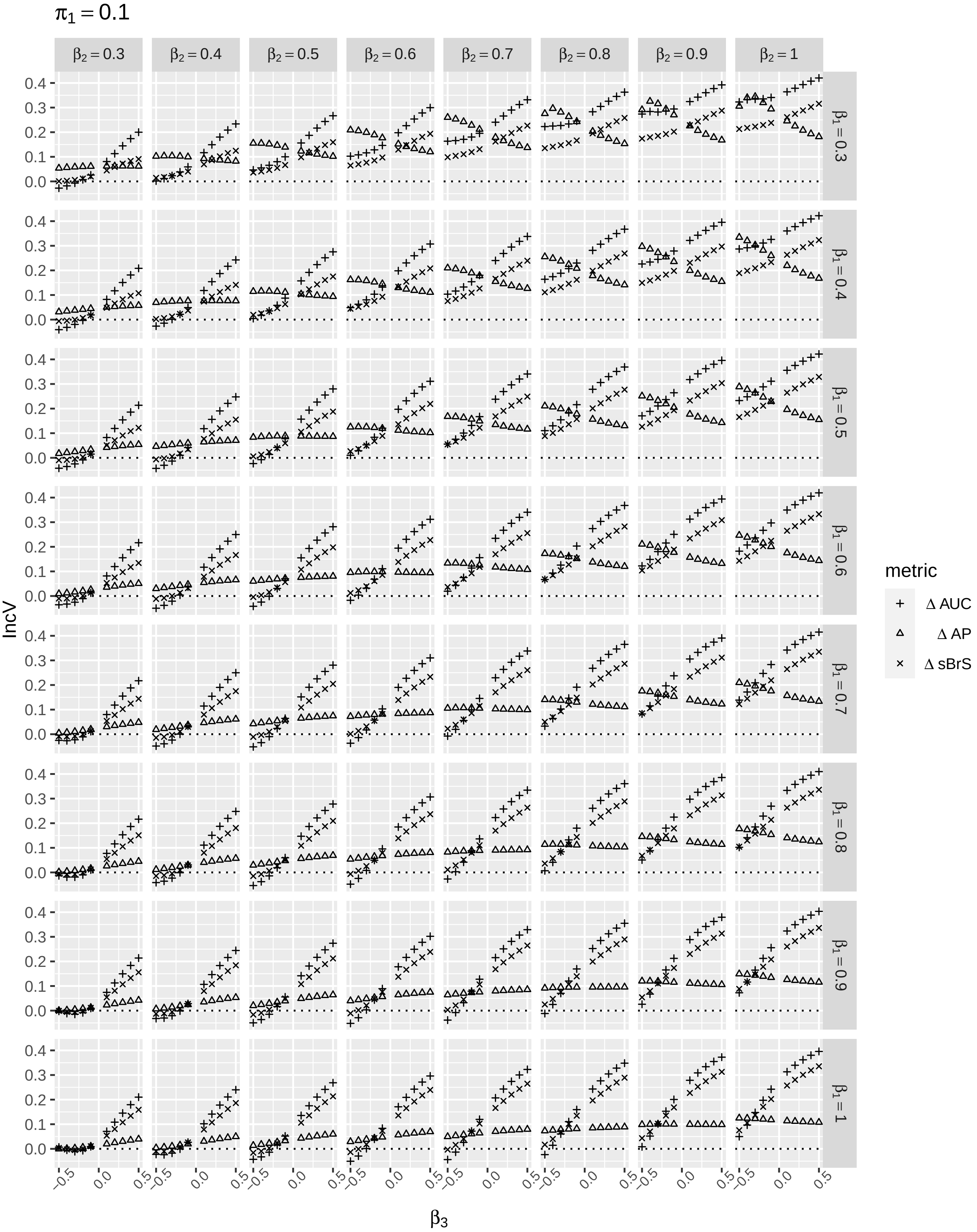

Figure S4: The ΔAUC, ΔAP, and ΔsBrS under the event rate $\pi = 0.1$.

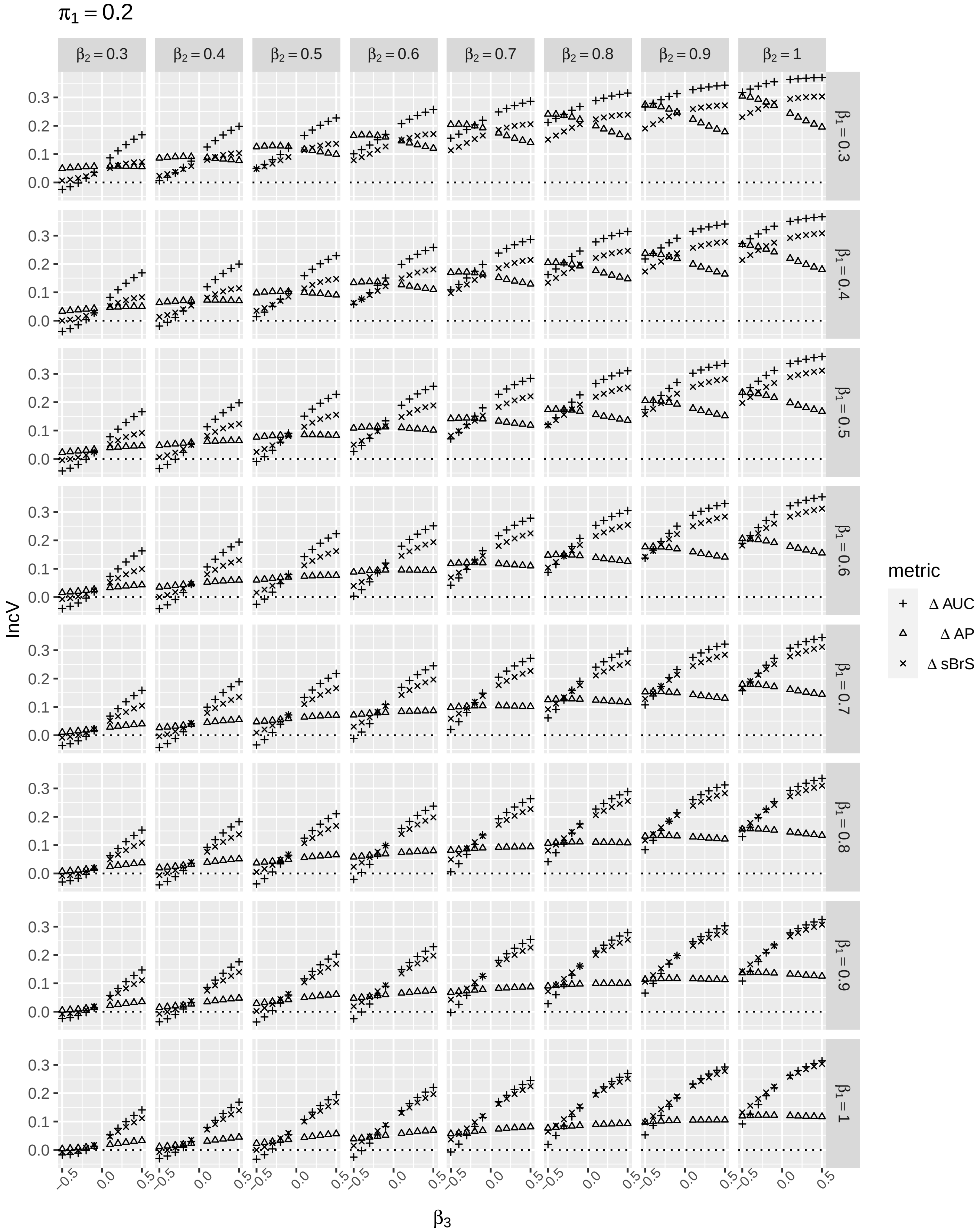

Figure S5: The ΔAUC, ΔAP, and ΔsBrS under the event rate $\pi = 0.2$.

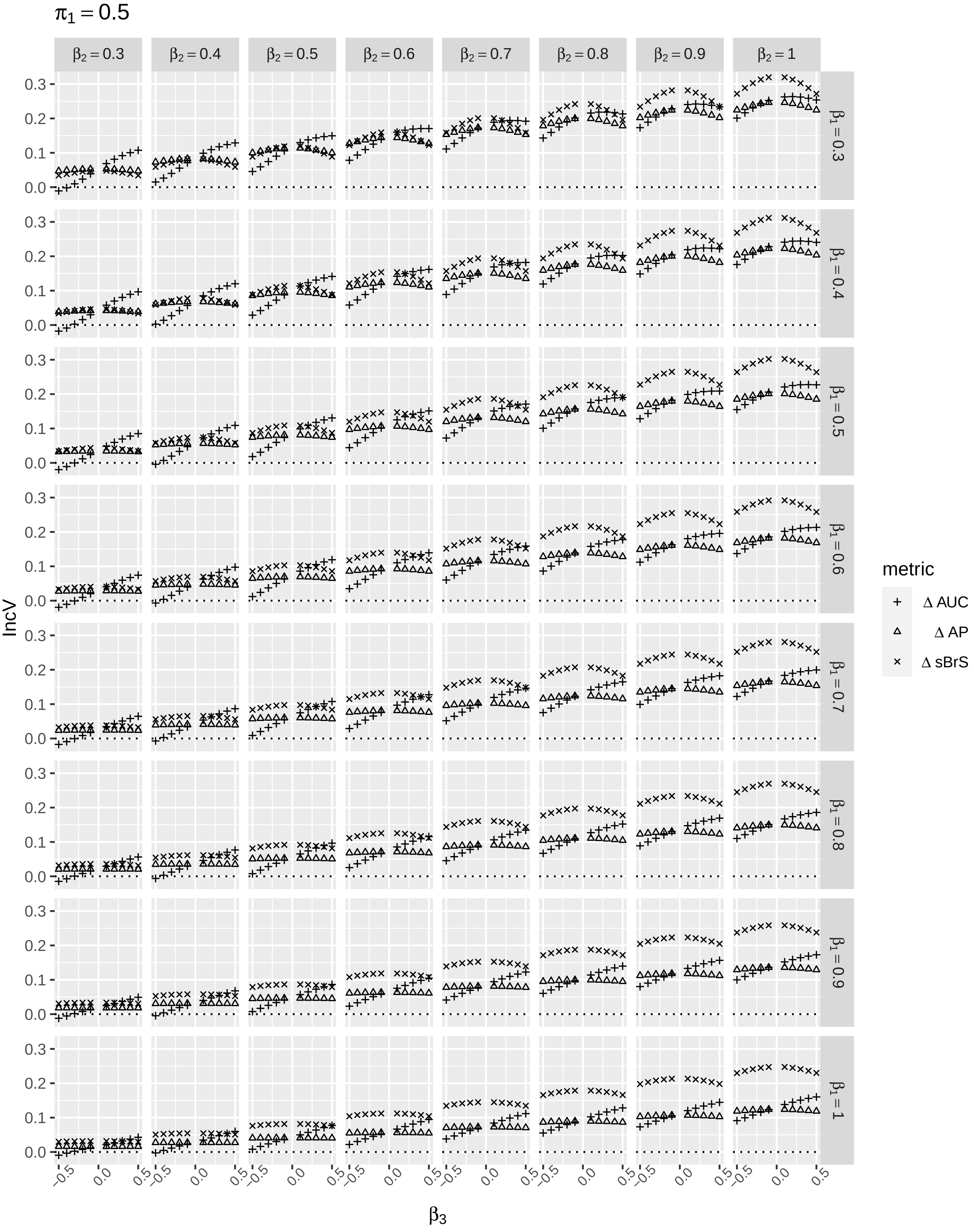

Figure S6: The ΔAUC, ΔAP, and ΔsBrS under the event rate $\pi = 0.5$.

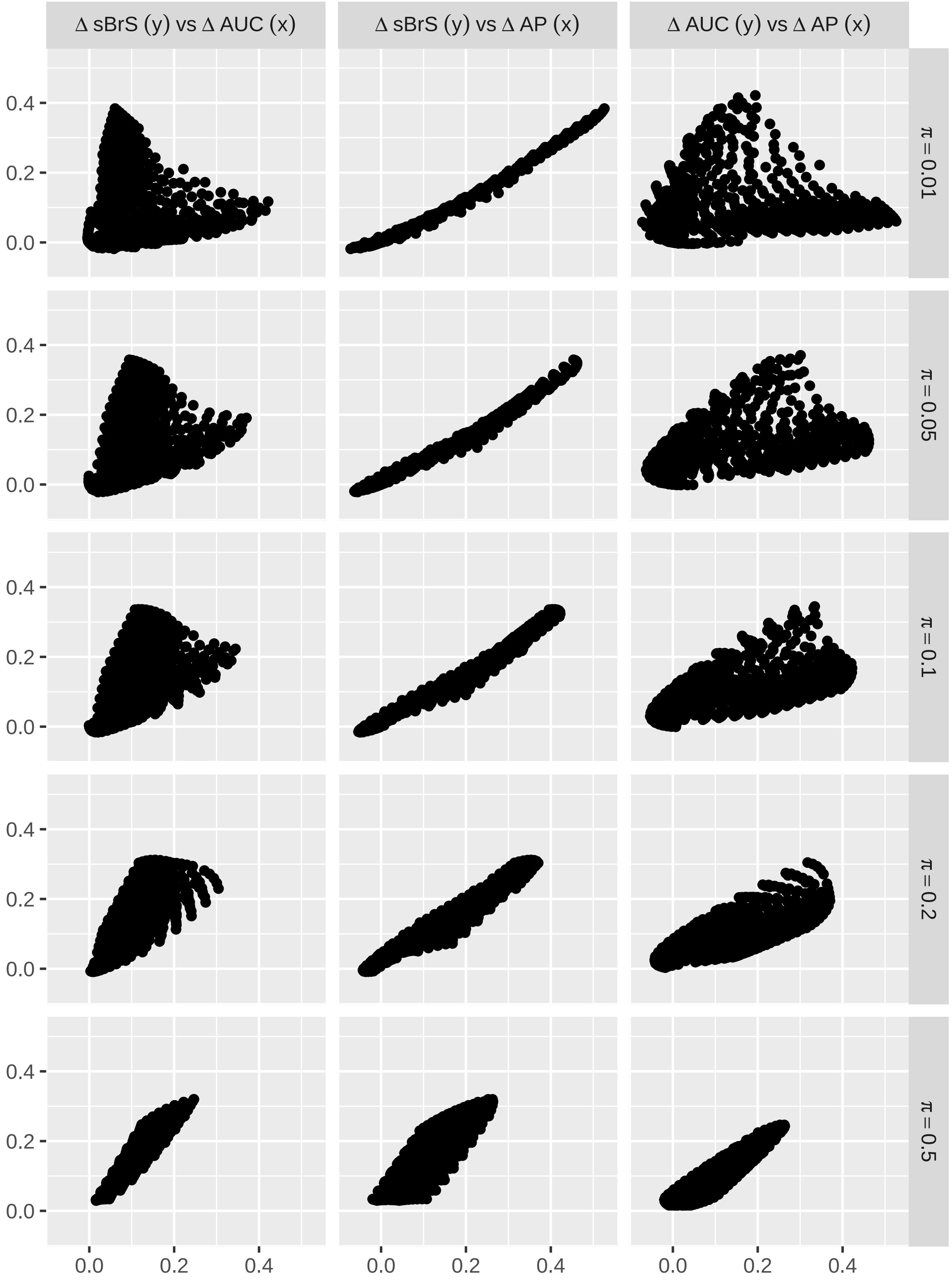

Figure S7: Scatter plots of each pair of the IncV metrics under different event rates π.

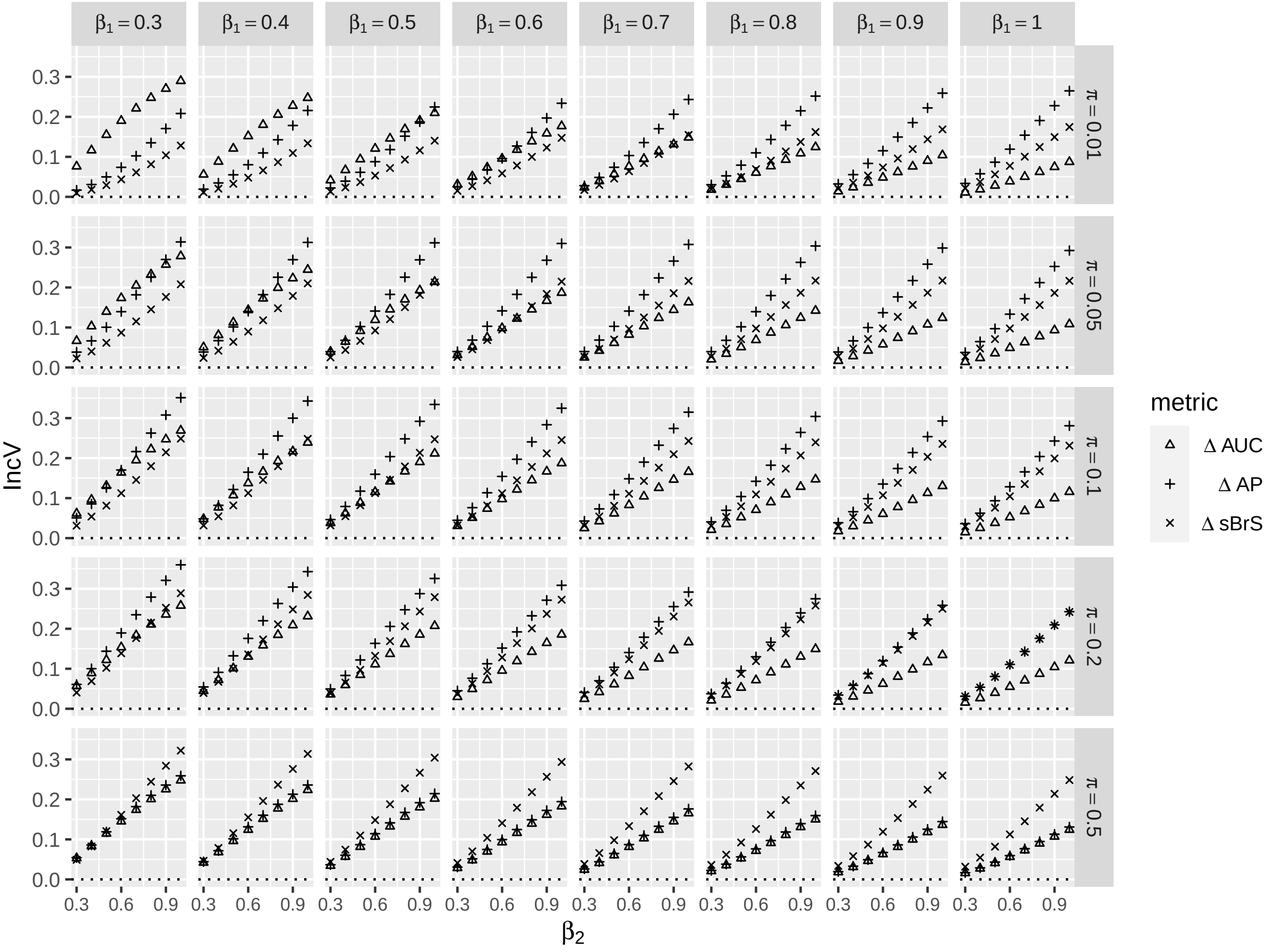

Figure S8: Plots of the values of each IncV metric under different event rates π when the two-marker model is the true model, i.e., $\beta_3 = 0$.

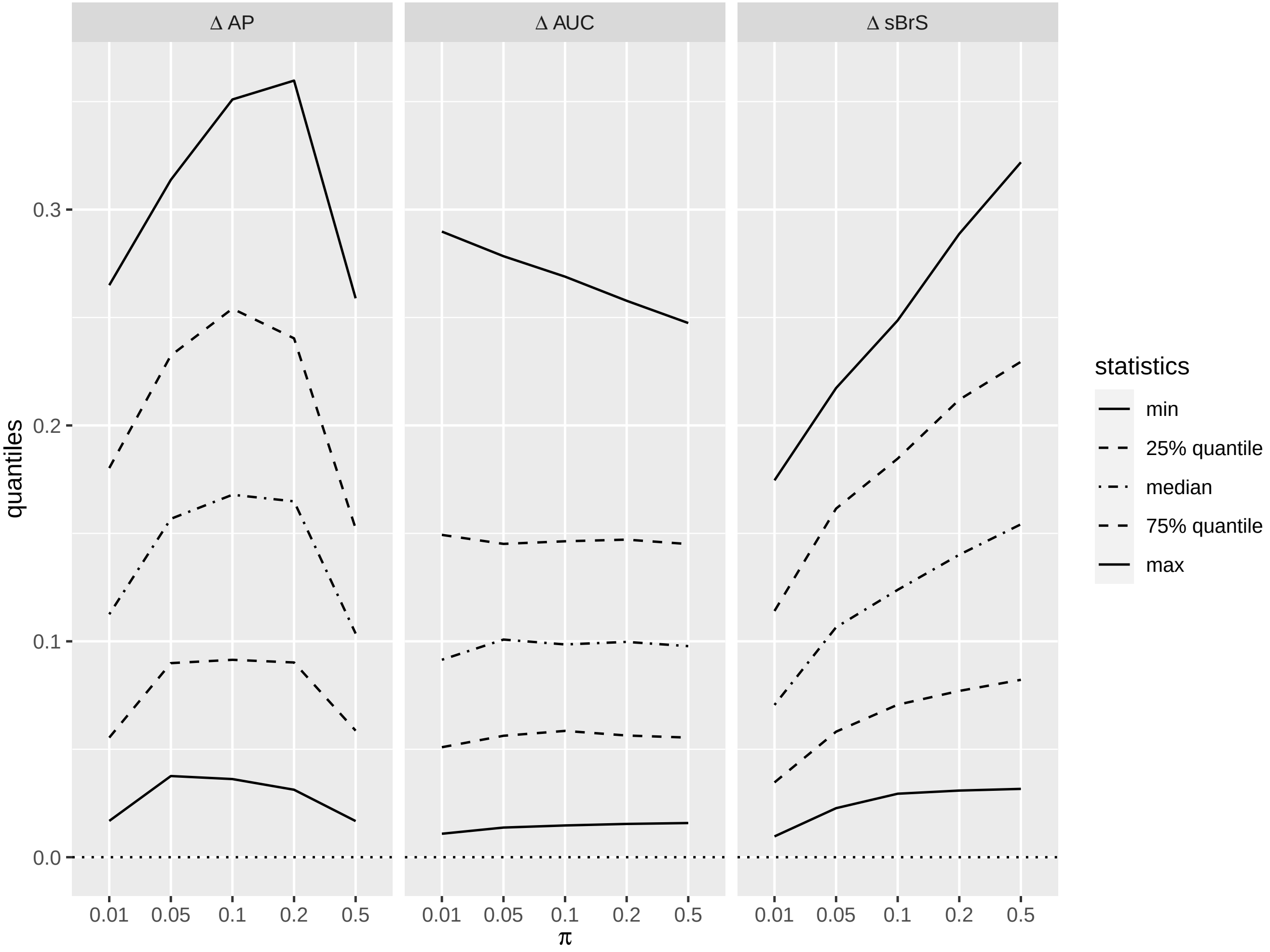

Figure S9: Summary statistics of each IncV metric versus different event rates π when the two-marker model is the true model, i.e., $\beta_3 = 0$. The statistics are the minimum, 25% quantile, median, 75% quantile, and maximum.

Table S1: Pearson correlation of each pair of the IncV metrics for different event rates π when the two-marker model is the true model, i.e., $\beta_3 = 0$.

Comparison	$\pi = 0.01$	$\pi = 0.05$	$\pi = 0.1$	$\pi = 0.2$	$\pi = 0.5$
ΔsBrS vs ΔAP	0.999	0.996	0.992	0.984	0.944
ΔsBrS vs ΔAUC	0.454	0.721	0.820	0.895	0.941
ΔAUC vs ΔAP	0.490	0.778	0.883	0.960	1.000

References

Borchers, H. W. (2019). *pracma: Practical Numerical Math Functions.*

Hasselman, B. (2018). *nleqslv: Solve Systems of Nonlinear Equations.*

R Core Team (2020). *R: A language and environment for statistical computing.* R Foundation for Statistical Computing.
